# The Interaction of *CircESR1* and HNRNPAB Regulates Cell Cycle Transition of Breast Cancer Cell

**DOI:** 10.7150/ijbs.126014

**Published:** 2026-01-14

**Authors:** Junchao Xu, Qiao Xu, Tingfang Cao, Miaomiao Wang, Yinzhong Shang, Junyan Tang, Sheng Wu, Xiaopeng Ma, Xinghua Han, Peter E. Lobie, Liting Qian, Tao Zhu

**Affiliations:** 1 Department of Oncology, The First Affiliated Hospital of USTC, State Key Laboratory of Immune Response and Immunotherapy, Center for Advanced Interdisciplinary Science and Biomedicine of IHM, Division of Life Sciences and Medicine, University of Science and Technology of China, Hefei, Anhui 230031, China.; 2 Institute of Cancer Research, Division of Life Sciences and Medicine, University of Science and Technology of China, Hefei, Anhui 230031, China.; 3 Anhui Key Laboratory of Molecular Oncology, Division of Life Sciences and Medicine, University of Science and Technology of China, Hefei, Anhui 230026, China.; 4 Shenzhen Bay Laboratory, Shenzhen, Guangdong 518055, China.; 5 Department of thyroid and breast Surgery, The First Affiliated Hospital of USTC, Center for Advanced Interdisciplinary Science and Biomedicine of IHM, Division of Life Sciences and Medicine, University of Science and Technology of China, Hefei, Anhui 230031, China.; 6 Institute of Biopharmaceutical and Health Engineering, Tsinghua Shenzhen International Graduate School, Tsinghua University, Shenzhen, Guangdong 518055, China.; 7 Department of Radiation Oncology, The First Affiliated Hospital of USTC, Center for Advanced Interdisciplinary Science and Biomedicine of IHM, Division of Life Sciences and Medicine, University of Science and Technology of China, Hefei, Anhui 230031, China.

**Keywords:** circRNA, HNRNPAB, estrogen receptor, cell cycle, therapeutic resistance, breast cancer

## Abstract

The mechanisms by which circRNAs regulate estrogen receptor (ER)-positive breast progression and therapeutic resistance remain poorly defined. By screening circRNAs involved in ER signaling, *circESR1* was identified as a novel circRNA exhibiting high specificity of expression in ER+ breast cancer. *CircESR1* interacted with HNRNPAB, which was transcriptionally activated by ER/SP1 signaling. HNRNPAB promoted the back-splicing and expression of *circESR1* by binding to the Alu elements of cognate pre-mRNA; and *circESR1* transcripts increased the stability and expression of HNRNPAB, ensuring an efficient positive feedback loop as reflected in antiestrogen-resistant breast cancer cells. Furthermore, HNRNPAB interacted and stabilized *CDK1* and *CDK6* mRNA, which was facilitated by its asymmetrical binding of *circESR1*, to promote cell cycle progression. Patients whose cancer exhibited high levels of *circESR1* and/or HNRNPAB exhibited advanced prognostic stage and poor survival. Combined use of *circESR1* ASO and CDK4/6 inhibitors were shown to be an effective therapeutic approach overcoming antiestrogen resistance in breast cancer xenograft models. Hence, these findings elucidated a novel signaling complex centered around *circESR1* and HNRNPAB in ER+ breast cancer, and suggested that *circESR1* might represent a potential therapeutic target for this disease.

## Introduction

Circular RNAs (circRNAs) have emerged as an important class of ncRNAs synthesized via the back-splicing of protein-coding genes 1, 2. Accumulating evidence suggests that circRNAs exert their functions by acting as decoys or sponges for microRNAs or proteins, as RNA scaffolds, or as translatable transcripts 3. CircRNAs are involved in multiple biological processes and diseases including cancer 4, 5, 6. The extraordinary stability exhibited by circRNAs due to their covalently closed circular structures renders them as promising candidate biomarkers for cancer diagnosis or prognosis 7, 8, 9, 10.

HNRNPAB belongs to the heterogeneous nuclear ribonucleoproteins (hnRNPs) subfamily, which have over 20 hnRNP members in humans, play a crucial role in controlling constitutive and alternative pre-mRNA splicing regulation in the nucleus as well as other aspects of mRNA metabolism and transport 11. A number of studies have revealed the roles of HNRNPAB as a nuclear protein in the regulation of proliferation, EMT and metastasis of various types of cancer cells by interacting with the 5'UTR or 3'UTR of mRNA, or the promoter DNA 12, 13, 14, 15, 16, 17. However, the mechanism and functions of HNRNPAB remain largely undescribed.

Estrogen receptor-α positive (ER+) breast cancer is a breast cancer subtype that accounts for at least two-thirds of all breast cancer 18. In ER+ breast cancer (BC), dysregulated ER signaling lead to aberrant expression of a large group of ER regulated genes with implications in cell cycle transition, cell death, and cell metabolism, among other functional considerations 19, 20, 21. Interestingly, higher expression of estrogen responsive genes such as BCL-2 22, GREB1 23 and ERLC1 24 correlate with better clinical outcomes in ER+ breast cancer, which reflects the opportunity for effective antiestrogen therapy for ER+ breast cancer patients. Endocrine therapies, including selective ER modulators (SERMs, e.g. tamoxifen), aromatase inhibitors (AIs), and selective ER down-regulators (SERDs, e.g. fulvestrant), may be utilized alone or in combination with CDK4/6 inhibitors (CDK4/6i, e.g. abemaciclib, palbociclib and ribociclib), for adjuvant therapy of ER+ BC patients. However, a large percentage of ER+ BC patients do not benefit from endocrine therapy due to primary resistance or acquired resistance after extended therapy 25, 26, 27, 28. Interestingly, 40-50% of ER+ patients that experience breast cancer relapse, still rely critically on ERα signaling for disease progression 29, 30. Tremendous effort has been exerted to identify mechanisms driving aberrant ER signaling in antiestrogen-resistant breast cancer cells including ERα modifications at either the genetic, epigenetic, or protein levels for sustained cell growth and/or cell survival 31, 32, 33. Recent work has shed light on ERα signaling associated circRNAs as well as their roles in ER+ breast cancer cells 34, 35, 36, 37, 38. Some of these circRNAs derived from the host genes which were part of ER signaling 35, 38. However, whether the pivotal genes of the ER signaling network, particularly the *ESR1* gene itself, could generate functional circRNAs in ER+ breast cancer remains uncharacterized.

In this study, *circESR1* was identified as a circRNA generated by back-splicing of the *ESR1* gene and which showed highly specific expression in ER+ breast cancer. It was observed that *circESR1* intertwined with ER signaling at multiple levels and promoted cell cycle transition and antiestrogen resistance via interaction with HNRNPAB. A novel therapeutic approach was subsequently proposed to specifically target *circESR1* in combination with CDK4/6 inhibitors to overcome endocrine therapy resistance.

## Materials and Methods

### Human tissue samples

Tissue specimens were obtained from 9 breast hyperplasia (benign), 13 ER- breast cancer, and 91 ER+ breast cancer patients treated at the First Affiliated Hospital of University of Science and Technology of China (Hefei, China). Specimens were encoded to protect the privacy and personal information of the patients. A summary of the clinical information of the patients is provided in Table S1. Sample collection was conducted in accordance with the Declaration of Helsinki ethical guidelines and approved by the Biomedical Ethics Committee of USTC (2020-P-054). The above tissues were only used for protein immunoblot, *in situ* hybridization and immunohistochemistry experimental research.

### Cell lines and culture

If not specified otherwise, all cell lines used in this study were obtained from American Type Culture Collection (ATCC). Cells were cryopreserved shortly upon receipt and continuously cultured for less than 2 months. The cell lines MCF-10A (ATCC CRL-10317), HMEC-hTERT (ATCC PCS-600-010), MCF-7 (ATCC HTB-22), T-47D (ATCC HTB-133), CAMA-1 (ATCC HTB-21), BT-474 (ATCC HTB-20), ZR-75-1 (ATCC CRL-1500), MDA-MB-231 (ATCC HTB-26), MDA-MB-468 (ATCC HTB-132), BT-549 (ATCC HTB-122), SK-BR-3 (ATCC HTB-30), Hs578T (ATCC HTB-126), 293T (ATCC CRL-3216) were authenticated by the analysis of short tandem repeat (STR) profiles and 100% matched the standard cell lines in the DSMZ data bank. These cells tested negative for cross-contamination of other human cells and mycoplasma contamination. HCC1937 (ATCC CRL-2336) and SUM149PT were from Dr. Ceshi Chen (KMU, China), SUM159PT was from Dr. Suling Liu (FDU, China), and HFL1 (ATCC CCL-153) was purchased from Procell Company (Wuhan, China).

MCF-7, T-47D, CAMA-1, BT-474, ZR-75-1, HCC1937 and BT-549 cells were cultured in RPMI-1640 (31800022, Gibco, Carlsbad, USA) supplemented with 10% FBS at 37℃ and 5% CO_2_. BT-549 cells were supplemented with 5 μg/mL insulin (Novolin R, Copenhagen, Denmark). SK-BR-3, Hs578T and 293T cells were cultured in DMEM supplemented with 10% FBS at 37℃ and 5% CO_2_. The culture of Hs578T cells was supplemented with 10 μg/mL insulin. MDA-MB-231 and MDA-MB-468 cells were cultured in Leibovitz's L-15 (41300039, Gibco, Carlsbad, USA) supplemented with 10% FBS at 37℃. SUM149PT and SUM159PT cells were cultured in Ham's F-12 (21700075, Gibco, Carlsbad, USA) supplemented with 5% FBS, 1 μg/mL hydrocortisone (A610506, Sangon Biotech, Shanghai, China) and 5 μg/mL insulin at 37℃ and 5% CO_2_. MCF-10A and HMEC-hTERT cells were cultured in DMEM/F-12 (12500062, Gibco, Carlsbad, USA) supplemented with 5% horse serum (E510006, Sangon Biotech Shanghai, China), 20 ng/mL EGF (AF-100-15, PeproTech, Cranbury, USA), 0.5 μg/mL hydrocortisone, 0.1 μg/mL CholeraToxin (CC104, Macgene, Beijing, China) and 10 μg/mL insulin at 37℃ and 5% CO_2_. HFL1 cells were cultured in Ham's F-12K (PM150910, Procell, Wuhan, China) supplemented with 10% FBS at 37℃ and 5% CO_2_. All medium contained 1% Pen-Strep solution (C0222, Beyotime Biotechnology, Shanghai, China). Estrogen deprivation and establishment of MCF-7 and T-47D TamR cells was as previously described 24.

### Mice

The animal experiment certification process was completed at the University of Science and Technology of China (PXHG-ZT201704275). All animal experiments were approved by the Institutional Animal Care and Use Committee, University of Science and Technology of China (USTCACUC24100122063). All surgery on experimental animals respected the rights of animals, and strictly met the SOP of the standard operating procedures for the Experimental Animal Center.

4-week-old female BALB/c nude mice were purchased from SPF Biotechnology Co. Ltd. (Beijing, China). All mice were maintained on a 12-hour light/dark cycle at 22-24 °C with 50-60% humidity, and with access to chow and water *ad libitum*. In xenograft models, 17β-Estradiol (2 μg per dose, T1048, Targetmol, Boston, USA) dissolved in 125 μL corn oil (ST2308, Beyotime Biotechnology, Shanghai, China) was injected subcutaneously every 3 days i.p. The xenograft volume was measured using a caliper every 3 days and was calculated as (length×width^2^)/2. To determine the function of *circESR1* or HNRNPAB depletion *in vivo*, 2×10^6^ *circESR1* or HNRNPAB depleted MCF-7 or control cells mixed 1:1 with Matrigel (BD Biosciences, New Jersey, USA) were injected into the second pair of fat pads on both sides of the mammary glands of female BALB/c nude mice (n=5/group). For xenograft assays to assess the effects of ASO or palbociclib treatment, 1×10^6^ MCF-7 TamR cells were orthotopically injected into the mammary fat pad of the female BALB/C nude mice (n=5/group). Tamoxifen (20 μg per dose, T6906, Targetmol, Boston, USA) dissolved in 125 μL corn oil was injected every 3 days i.p. When the xenograft volume reached approximately 200 mm^3^, xenograft-bearing mice were randomized and received intratumoral injection of negative control or ASO-*circESR1* (5nM per dose, every 3 days, RiboBio, Guangzhou, China) in the presence or absence of palbociclib (100mg/kg/week i.g., T1785, Targetmol, Boston, USA). Mice were sacrificed simultaneously when xenografts reached 600 mm^3^ and the dissected xenografts were processed for further histological analysis.

### Plasmid constructions and reagents

shRNAs for *circESR1* depletion were obtained from CircInteractome website (https://circinteractome.nia.nih.gov/) and synthesized by Sangon Biotech (Shanghai, China). For *circESR1* forced expression plasmid pCDH-circ 39, 40, two tandem reverse complementary intron sequences from a commercial vector pLCDH-ciR of Geneseed Biotech (Guangzhou, China) 41, 42, 43 were inserted into pCDH-CMV-MCS-EF1-Puro (SBI). The mature *circESR1* sequence was PCR amplified and inserted into the region between the two introns to provide AG receptor and GT donor pairing. Cyclization was confirmed by divergent primers and SJOD primer (Splice Junction Overlapping Divergent Primers) and the accuracy of conjunction confirmed by Sanger sequencing.

The shRNA plasmids for various human genes were obtained from The RNAi Consortium (MISSION® TRC shRNA library, Sigma-Aldrich, Darmstadt, Germany). The sgRNAs targeting exon 2 of human HNRNPAB were designed based on https://www.benchling.com/crispr. After transfection of lentiCRISPRV2 plasmids expressing Cas9 mRNA and targeted sgRNAs for two days, cells were selected by puromycin for a week. Cells were seeded in monoclonal form and slowly grown in 96-well plates, and then immunoblot was performed to verify knockout efficiency. For luciferase reporter plasmids of HNRNPAB promoters, the DNA fragments upstream of HNRNPAB gene carrying transcription factor binding sites were cloned into the pGL3-Basic plasmid (Promega, Wisconsin, USA). The coding sequence of HNRNPAB (Homo) genes were amplified and sub-cloned into the pCDH-3xflag vector to generate expressing plasmid. All constructs were verified by DNA sequencing. The sequences of shRNAs and primers for cloning are listed in Table S2-S3.

Actinomycin D (HY-17559), Cycloheximide (CHX, HY-12320), MG-132 (HY-13259) and Propidium Iodide (PI, HY-D0815) were from MedChemExpress (New Jersey, USA). Tamoxifen (T6906), Fulvestrant (T2146), Abemaciclib (T2381), Palbociclib (T1785) and Ribociclib (T6199) were from Targetmol (Boston, USA).

### Cell transfection and lentiviral transduction

For transfection of siRNA or ASOs, cells were plated at 5 × 10^4^ per well in 12-well plate and transfected with specific siRNAs (100 nM, Generalbiol, Anhui, China) or ASOs (50 nM, RiboBio, Guangzhou, China) mixed with Lipofectamine 2000 (Invitrogen, Carlsbad, USA) according to the manufacturer's instructions. The sequences of siRNAs are listed in Table S4.

For stable transduction, the shRNAs and control viruses were generated by transfection of corresponding plasmids together with the pGag/Pol, pRev and pVSV-G plasmids into 293T cells using Polyethyleneimine (PEI, 23966-1, Polysciences, Pennsylvania, USA). Additionally, the forced expression, sgRNAs and control viruses were generated by transfection of corresponding plasmids together with the pMD2.G and psPAX2 plasmids into 293T cells using PEI. The virus particles were harvested at 48 and 72 h later and filtered by a 0.22 μm filter unit (Filter-bio, Nantong, China). MCF7 cells were infected with recombinant retrovirus-transducing units in the presence of 8 μg/mL Polybrene (H9268, Sigma-Aldrich, Darmstadt, Germany) for 24 h and then selected with 2 μg/mL puromycin (A610593, Sangon Biotech, Shanghai, China). The sequences of sgRNAs are listed in Table S5.

### Cell function assays and flow cytometry analysis

2,000 cells/well were seeded into 96-well plates in complete or low serum (1%) medium for the determination of cell viability by MTT (A600799, Sangon Biotech, Shanghai, China) assay. To evaluate the ability of foci formation, 5,000 cells were seeded into 6-well plates and cultured in complete medium at 37℃ for 2 weeks. Colonies were stained using 1% crystal violet. All experiments were repeated three to five times.

For cell cycle analysis, cells were collected, fixed with 70% (v/v) ice cold ethanol overnight at -20 ℃, rinsed twice with PBS, and then stained with PBS containing 50 µg/mL PI, 100 µg/mL RNase A, and 0.2% Triton X-100 for 30 min. For cell death analysis, the apoptotic cells were examined using Annexin V-FITC/PI kit (V13245, Invitrogen, Carlsbad, USA). The FACS analysis was performed on a BD Fortessa Flow Cytometer.

### Total RNA extraction, qRT-PCR, RNase R treatment and RNA-seq analysis

Total RNA was extracted using TRIzol reagent (GK20008, GlpBio, California, USA), and cDNA reverse transcribed with TransScript® All-in-one First-Strand cDNA Synthesis SuperMix for qPCR (AT341, TransGen Biotech, Beijing, China). qRT-PCR was performed using Stratagene Mx3000P (Agilent, California, USA) with TransStart® Tip Green qPCR SuperMix (AQ141, TransGen Biotech, Beijing, China). The sequences of primers for qRT-PCR are listed in Table S6. The quantification of mRNA expression was obtained with the 2^-ΔΔCt^ method. For RNase R digestion experiments, RNA samples were incubated 30 min at 37℃ with 3 U/μg of RNase R (RNR07250, Epicentre, Wisconsin, USA).

Total RNA was extracted and the quantified and samples with OD 260/280 ratio > 1.8 and OD 230/260 ratio > 2 were sent to BGI Genomics (Shenzhen, China) for sequencing. Gene Ontology (GO) enrichment analysis was performed using the clusterProfiler package in R. *P value* was adjusted using the Benjamini-Hochberg method to obtain *Q value* (FDR-corrected *p-value*). Terms with *Q value* < 0.05 were considered significantly enriched. The datasets were then sorted based on the number of candidate genes they contained in descending order, and the top 20 datasets were selected for display. To identify enriched functional pathways, Gene Set Enrichment Analysis (GSEA) was performed on the ranked gene list, determined by the Log_2_ Fold-Change.

### Immunoblot assay

Whole cell lysate was prepared using RIPA lysis buffer (P0013, Beyotime Biotechnology, Shanghai, China) with 1 mM PMSF, protease inhibitors and phosphatase inhibitor cocktail (P1050, Beyotime Biotechnology, Shanghai, China). Protein concentration was determined by bicinchoninic acid (BCA) assay (P0012, Beyotime Biotechnology, Shanghai, China). Equal amounts of protein lysates were resolved by SDS-PAGE gels and then transferred on a NC membrane (Millipore, Darmstadt, Germany). After overnight incubation with primary antibody (listed in Table S7), the membranes were hybridized with a 1:10,000 dilution of peroxidase-conjugated goat anti-mouse or anti-rabbit antibody (115-035-003, 111-035-003, Jackson ImmunoResearch, Pennsylvania, USA) at room temperature for 1 h. The immunoreactive signals were visualized by SuperSignal™ West Pico PLUS Chemiluminescent Substrate (34580, Thermo Fisher, Carlsbad, USA) and Tanon 4160 Automatic Chemiluminescence Image Analysis System.

### Digital (d)PCR

dPCR experiments were constructed on the Clarity^TM^ Digital PCR system (Singapore) using BioDigital Maxuseful dPCR Kit (ARX-004, Saint Genomics, Jiangsu, China). Reaction mix containing either 8.3 µL cDNA or water (no-template controls) were prepared by adding 5 µL 3× Maxuseful dPCR Buffer, 0.5 µL Taq DNA polymerase, 0.6 µL 10 µM forward primer and 0.6 µL 10 µM reverse primer. Chips were run on the LongGene PCR System T20 (Hangzhou LongGene Scientific Instruments Co., Ltd.) by applying the following conditions: hot-start at 50 ℃ for 5 min; hold at 95℃ for 5 min; followed by 50 cycles of 95 ℃ for 10 s and 60 ℃ for 30 s; hold at 25 ℃. The data was analyzed by Clarity™ software (Singapore). *CircESR1* relative quantification was obtained by normalization of its expression level (copies/µL) on the number of MCF-7 and T-47D cells.

### Polysome profiling

The polysome profiling assay was carried out as previously described 44. A total of 10^7^ cells were treated with ice-cold phosphate buffered saline or culture medium containing 100 mg/mL cycloheximide (Sigma-Aldrich, Darmstadt, Germany) for 10 min followed by lysis in ribosome lysis buffer (10 mM Tris·HCl [pH 7.4], 5 mM MgCl_2_, 100 mM KCl, 1% Triton X-100, 3 mM dithiothreitol [DTT], 100 mg/mL cycloheximide, 5 U/mL RNase inhibitor [Promega, Wisconsin, USA], and 1×Protease-inhibitor mixture [Roche]). Polysomes were separated on a 20 to 50% linear sucrose gradient containing 20 mM Tris (pH 7.4), 5 mM MgCl_2_, 100 mM KCl, 3 mM DTT, 100 mg/mL cycloheximide, and 1 U/mL RNase inhibitor (Promega, Wisconsin, USA) and centrifuged at 38,000 rpm for 4 h in a Beckman SW41 Ti rotor. The curve was generated with optical scanning at 254 nm using a Gradient Profiler (BioComp, Canada).

### Biotin RNA pull-down

Breast cancer cells were lysed in lysis buffer (150 mM NaCl, 50 mM Tris, 5 mM EDTA, 1% NP-40, 0.1% SDS, 1 mM dithiothreitol (DTT), 0.1U/μL RNase inhibitors, 1× protease inhibitors, 1 mM PMSF). The sample were rotated for 30 min at 4℃ and sonicated. The cell lysates were centrifuged 12,000 rpm for 10 min. The supernatant was collected and precleared with M-280 Streptavidin Dynabeads (11206D, Invitrogen, Carlsbad, USA) at 4℃ for 2h. Afterwards, the supernatant was collected, 200 pmol of biotin-DNA oligonucleotides were added and incubated at 4 ℃ overnight. M-280 Streptavidin Dynabeads were pretreated three times in the lysis buffer, blocked with 500 ng/μL yeast RNA and 1 mg/mL BSA for 2 hours at room temperature, and then washed three times with lysis buffer. The treated beads were then added into the samples with rotation for 4 hours at 4 °C. Beads were captured with magnets, washed twice with lysis buffer, and thrice with lysis buffer supplemented with 500 mM NaCl. The enrichment of *circESR1* in the capture fractions was evaluated by qRT-PCR analysis. The bound proteins were eluted from the packed beads and analyzed by SDS-PAGE. The proteins in the capture complex were identified by western blot, silver staining, or mass spectrometry analysis. The sequences of pull-down probes are listed in Table S8.

### RNA-binding protein immunoprecipitation (RIP) and RIP-seq analysis

RIP assay was performed according to the manufacturer's protocol using the EZ-Magna RIP™ Kit (17-701, Millipore, Darmstadt, Germany). RNA was purified after proteinase K digestion and extracted using TRIzol reagent (GK20008, GlpBio, California, USA). The samples from the input group and the anti-HNRNPAB antibody enrichment group were sent to Aksomics (KangChen Bio-tech, Shanghai, China) for RIP-seq.

### Luciferase reporter assays

MCF-7 cells were seeded at 60% confluence in 24-well plates. For transcription factor mediated HNRNPAB expression, 0.2 μg pGL3 Basic luciferase reporter (RRID: Addgene_48743), 50 nmol JUN or FOS or FOXA1 or SP1 siRNAs and 0.02 μg pRL-TK plasmid (RRID: Addgene_11313) were transfected into cells using Lipofectamine 2000 (Invitrogen, Carlsbad, USA). The pRL-TK plasmid was provided as an internal transfection control. The total cell lysates were harvested 48 h after transfection, and the firefly and Renilla luciferase activities were measured using the Dual-Luciferase reporter assay system (Promega, Wisconsin, USA) according to the manufacturer's instructions.

### Northern blot

The DIG-labeled short back-splicing probe specific to *circESR1* used in Northern experiments was purchased from Sangon Biotech (Shanghai, China). The sequences of Northern probes are listed in Table S9. Total RNA was extracted from MCF-7 cells with standard TRIzol methods. A total of 8% tris-borate EDTA (TBE)-urea polyacrylamide gel electrophoresis with 8 M urea was prerun for 2 hours. Then, 20 μg of RNA and RiboRuler High Range RNA Ladder (Thermo Fisher, Carlsbad, USA) were loaded on prerun polyacrylamide gel and run for another 2 hours in 0.5 × TBE buffer. RNA in polyacrylamide gel was transferred onto Hybond-N+ membranes (Millipore, Darmstadt, Germany) at 4 ℃ overnight. After crosslinking using an ultraviolet light, hybridization was performed at 60 ℃ overnight. Detection was performed according to the manufacturer's instructions (DIG Northern Starter Kit, 12039672910, Roche, Basel, Switzerland). Images were taken with Tanon 4160 Automatic Chemiluminescence Image Analysis System.

### Immunofluorescence

Breast cancer cells grown on 15 mm cell climbing tablets (801007, NEST, Jiangsu, China) in 24-well plates were fixed with 4% paraformaldehyde in PBS for 15 min. After washing three times with PBS, the cells were permeabilized with PBS containing 0.25% Triton X-100 for 5 min. The cells were washed three times with PBS and blocked with 1% BSA for 1 h. Then the samples were incubated in a wet box with primary antibody overnight at 4℃. After using PBST to wash three times, the samples were incubated with corresponding secondary antibody for 1 h at room temperature, followed by staining with DAPI (1 μg/mL) for nucleus staining. Fluorescent images were acquired using a fluorescence microscopy (ZEISS LSM980 confocal microscopy, Germany). The relative fluorescence densities were analyzed by ImageJ.

### RNA fluorescence *in situ* hybridization (FISH)

The Cy3-labeled short back-splicing probes specific to *circESR1* used in FISH were purchased from Ruibo Biotechnology Co., Ltd (Guangzhou, China). Cells were fixed with PBS containing 10% methanol and acetic acid (3:1) for 10 min. The samples were dehydrated with 70, 90 and 100% ethanol, followed by prehybridization (550 μL formamide, 324 μL DEPC water, 50 μL 20× SSC, 0.1 g Dextran sulfate, 25 μL 10 mg/mL yeast RNA, 50 μL 10 mg/mL sheared salmon sperm DNA, 1 μL RNase inhibitor) at 37℃ for 2 h. Samples were hybridized with labelled RNA probes in hybridization buffer at 37 °C overnight in a dark wet box. After being washed four times in 50% formamide/2× SSC for 5 min at 45℃ and three times in 2× SSC for 5 min at 45℃, the samples were incubated with DAPI. Images were acquired using fluorescence microscopy (ZEISS LSM980 confocal microscope, Germany). Pearson's correlation coefficient was analyzed by ZEISS ZEN 3.8 software.

### Immunohistochemistry (IHC) and *in situ* hybridization (ISH)

Paraffin-embedded tissue sections were de-paraffinized in xylene and rehydrated through graded ethanol. After antigen retrieval by boiling in 10 mM sodium citrate buffer (pH 6.0) for 90 sec, endogenous peroxidase blocking buffer (SP KIT-A2, MXB Biotechnologies, Fuzhou, China) was added for 10 min. Tissues were washed three times with PBST for 5 min and then incubated with anti-HNRNPAB (1:1,000, ab199724, Abcam, Cambridge, UK) antibodies at 4 ℃ overnight. Immunostaining was performed using the UltraSensitive S-P Detection Kit (KIT-9720, MXB Biotechnologies, Fuzhou, China), and color was developed by using a DAB kit (ZLI-9018, ZSGB-BIO, Beijing, China). Subsequently, sections were counterstained with hematoxylin. The DIG-labeled short back-splicing probe specific to *circESR1* used in ISH experiments was purchased from Sangon Biotech. ISH assay was performed with the Enhanced Sensitive ISH Detection Kit I (POD, MK1030, Boster Biological Technology, Wuhan, China) according to the manufacturer's instructions. Immunohistochemistry and in situ hybridization images were taken using an Olympus inverted fluorescence microscope IX73 (Olympus, Japan). Following digital scanning, images of serial tissue sections were acquired using CaseViewer software (3DHISTECH, Hungary).

The staining scores were determined by two independent observers, based on both the proportion and labeling intensity of the HNRNPAB protein or *circESR1* positive cells. The proportion of positively stained tumor cells was divided into 5 grades: (0: < 5%; 1: 5-25%; 2: 26-50%; 3: 51-75%; and 4: > 75%). The staining intensity was recorded as follows: 0 (no staining), 1 (light brown), 2 (brown), and 3 (dark brown). The SI was calculated as follows: SI = the proportion of positive cells × staining intensity. Using this method, the expression of target protein was evaluated using the SI and scored as (0, 1, 2, 3, 4, 6, 8, 9, or 12), with a cut-off point of < 6 versus ≥ 6.

### Statistics and reproducibility

Statistical analyses were performed using GraphPad Prism 10.0 software. All *in vitro* and animal experiment results are presented as means ± standard deviation (S.D.) and two-tailed Student's test were used to calculate the *p* value. Survival curves were constructed using K-M analysis and compared using two-tailed log-rank test. Chi-square test was used for variable comparison, with *p* < 0.05 regarded as statistically significant. Spearman's method was used to assess the correlation between factors. *p* < 0.05 was regarded as statistically significant. All experiments were biologically repeated at least three times.

## Results

### Specific expression of *circESR1* in ER+ breast cancer

**Figure 1:**
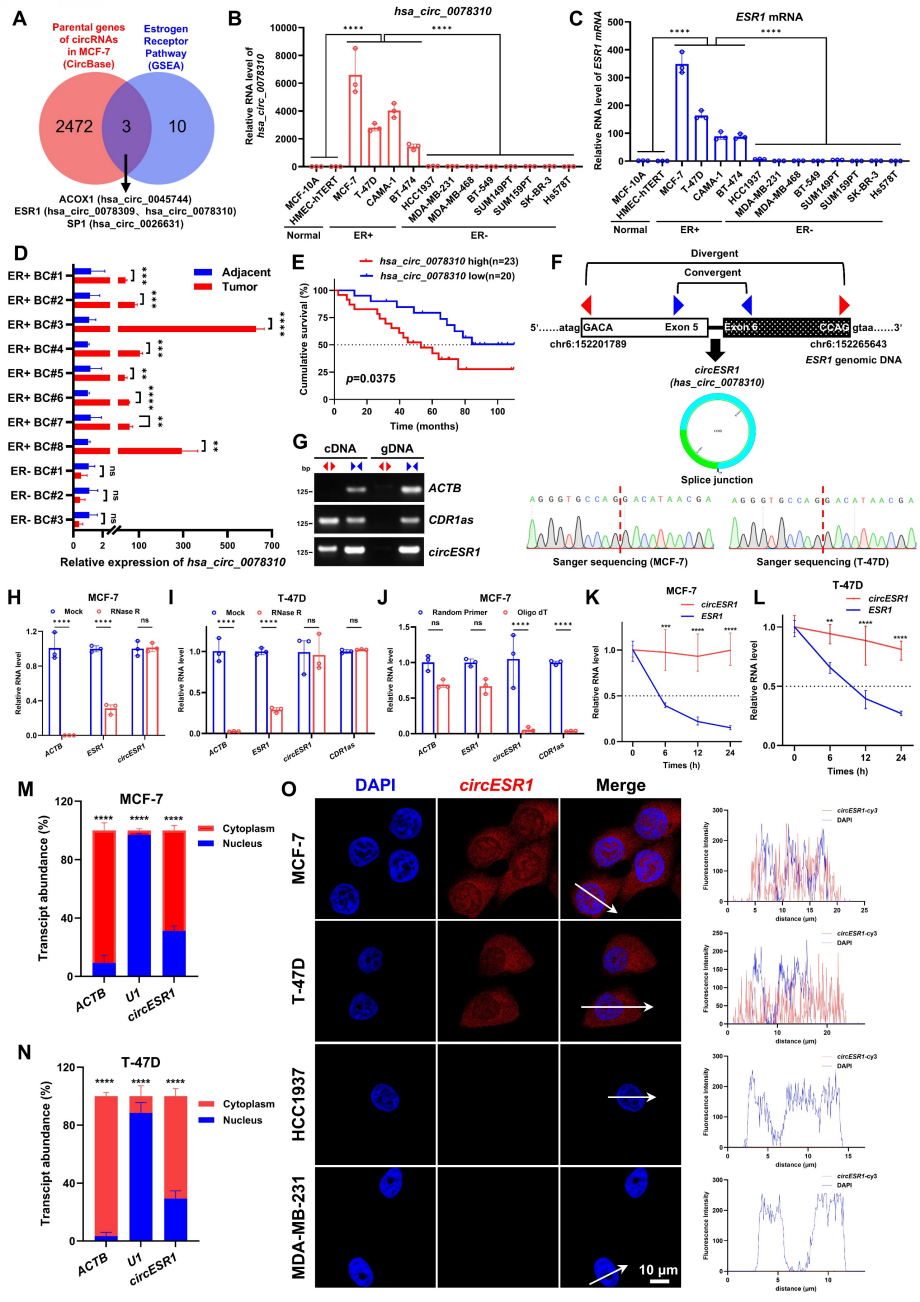
** Identification of *circESR1* in ER+ BC.** (A) Venn diagram revealing the intersection of 2,475 parental genes of 5,331 circRNAs against 13 genes in estrogen receptor pathway. The 13 estrogen receptor pathway genes we selected were from GSEA dataset with source number WP2881 (WP-ESTROGEN-RECEPTOR-PATHWAY, https://www.wikipathways.org/pathways/WP2881). (B-C) The relative expression of *hsa_circ_0078310* or *ESR1* mRNA in 2 normal breast epithelial cell lines, 4 ER+ BC cell lines and 8 ER- BC cell lines analyzed by qRT-PCR. (D) The relative expression of *hsa_circ_0078310* in tumor tissues and matched adjacent normal tissues derived from 8 ER+ and 3 ER- BC patients analyzed by qRT-PCR. (E) Kaplan-Meier analysis of OS in ER+ BC patients with low versus high expression of *hsa_circ_0078310*. n=43, *P* value was determined by two tailed log-rank test. (F) Head-to-tail splicing junction in *circESR1* with cDNA from MCF-7 and T-47D cell lines analyzed by Sanger sequencing. (G) *CircESR1*, along with *ACTB* and circular RNA *CDR1as*, was amplified from cDNA or gDNA from MCF-7 cells with divergent and convergent primers, respectively. (H-I) The relative expression changes of *ACTB* mRNA, *ESR1* mRNA, *circESR1*, and *CDR1as* in MCF-7 or T-47D cells after RNase R digestion treatment analyzed by qRT-PCR. (J) The relative expression changes of *ACTB* mRNA, *ESR1* mRNA, *circESR1*, and *CDR1as* in MCF-7 cells after reverse transcription using random primers and Oligo(dT) analyzed by qRT-PCR. (K-L) The relative expression changes of *ESR1* mRNA and *circESR1* in MCF-7 and T-47D cells treated with actinomycin D for 24 hours analyzed by qRT-PCR (n=3 for each time point). (M-N) After nuclear and cytoplasmic separation of MCF-7 and T-47D cells, the relative expression levels of *ACTB* mRNA, *U1* RNA, and *circESR1* analyzed by qRT-PCR. (O) Immunofluorescence analysis of DAPI (blue) and *circESR1* AS probe labeled with cy3 (red) in MCF-7, T-47D, HCC1937 and MDA-MB-231 cells. Scale bars, 10 μm. Data was shown as mean ± S.D. from three independent experiments. Unpaired two-tailed Student's *t* test (D, H-N) and one-way ANOVA followed by Tukey's multiple comparisons test (B-C). ns, *P*>0.05; *, *P*<0.05; **, *P*<0.01; ***, *P*<0.001; ****, *P*<0.0001.

Whereas host gene-derived circRNAs generally regulate cellular processes independently of their parental genes, a subset may functionally align with host gene activity 45, 46, 47, 48. To explore the circRNAs contributing to estrogen receptor signaling, we selected an array of functionally relevant host genes that possess potential for generating circRNAs (Figure 1A and Figure S1A). A matrix of 13 genes known to function in ER signaling (source number WP2881) was subsequently determined based on gene set enrichment analysis (GSEA). Using the circBase data from Salzman et al. 49, which detailed 5,331 validated human circRNAs, we cross-referenced their corresponding 2,475 parental genes against the 13 genes, deriving a prioritized list of three host genes associated with ER signaling, namely *ACOX1*, *ESR1* and *SP1* (Figure 1A and Figure S1A-B). According to the circBase, the *ESR1* gene was predicted to generate two distinctive circRNAs, i.e. *hsa_circ_0078309* and *hsa_circ_0078310* (Figure S1C-E). Moreover, ACOX1 and SP1 genes were predicted to generate only one circRNA respectively, i.e. *hsa_circ_0045744* (parent gene *ACOX1*) and *hsa_circ_0026631* (parent gene *SP1*) (Figure S1C). In an array of mammary epithelial and carcinoma cells (MCF-10A, HMEC-hTERT, MCF-7, T-47D, MDA-MB-231 and SUM159PT), we analyzed the expression of four circRNAs and observed that only the expression of *hsa_circ_0078310* was highly elevated in two ER+ BC cell lines compared to two normal or two ER- BC cells (Figure S1F).

It was interesting to observe the highly specific expression of *hsa_circ_0078310*, along with its parental gene *ESR1*, in four ER+ BC cells compared to two normal or eight ER- BC lines (Figure 1B-C). By using tumor tissues and matched adjacent normal tissues derived from eight ER+ and three ER- BC patients, elevated expression of *hsa_circ_0078310* was observed in all ER+ tumor tissue samples compared to the adjacent non-tumor or ER- BC tissue samples (Figure 1D). IHC and ISH staining using serial tissue sections derived from 91 paraffin-embedded ER+ BC specimens showed that elevated expression of *hsa_circ_0078310* correlated with high ER level, advanced stage and Ki-67 positivity (Table 1 and Figure S8A). Prognosis analysis based on ISH staining using 43 paraffin-embedded ER+ BC specimens revealed that higher *hsa_circ_0078310* expression correlated with worse overall survival in ER+ BC patients (Figure 1E). *hsa_circ_0078310* was designated as *circESR1* hereafter, according to the naming guideline for circRNAs 50.

According to circBase dataset, *circESR1* is a 453-nt circRNA generated by back-splicing of exons 5 and 6 of the *ESR1* gene located on human chromosome 10 with no homology to murine sequences 49. Sanger sequencing validated that its head-to-tail splice junction region was identical to the reported sequence in MCF-7 and T-47D cells (Figure 1F). Consistent with the circular form, the divergent primers for *circESR1* and *CDR1as*, but not *ACTB*, amplified a PCR product respectively 51 (Figure 1G). Resistance to digestion with RNase R exonuclease (Figure 1H-I) and inability to be reverse transcribed by Oligo (dT) (Figure 1J) indicated high stability of this RNA species. The half-life of *circESR1* (> 24 h) was significantly greater than that of the cognate linear transcript (~6 h) in ER+ BC cells (Figure 1K-L). It was estimated by digital (d)PCR that ~233 and ~128 copies of* circESR1* per cell exist in MCF-7 and T-47D cells, respectively (Figure S1G-H). Northern blot analysis confirmed that *circESR1* resolved at ~500 nt (Figure S1I). Furthermore, *circESR1* was localized in both the cytoplasm and nucleus as observed by subcellular fractionation (Figure 1M-N) and FISH analysis (Figure 1O). Polysome profiling assays in MCF-7 cells showed that compared to *GAPDH* mRNA, neither *circHIPK3* nor *circESR1* were appreciably associated with low-molecular-weight (LMW) nor high-molecular-weight (HMW) polysomes (Figure S1J-K), suggesting that *circESR1* possesses no apparent protein-coding potential. Together, these data established that *circESR1* harbors a circular RNA structure.

### *CircESR1* promotes cell cycle transition

**Figure 2:**
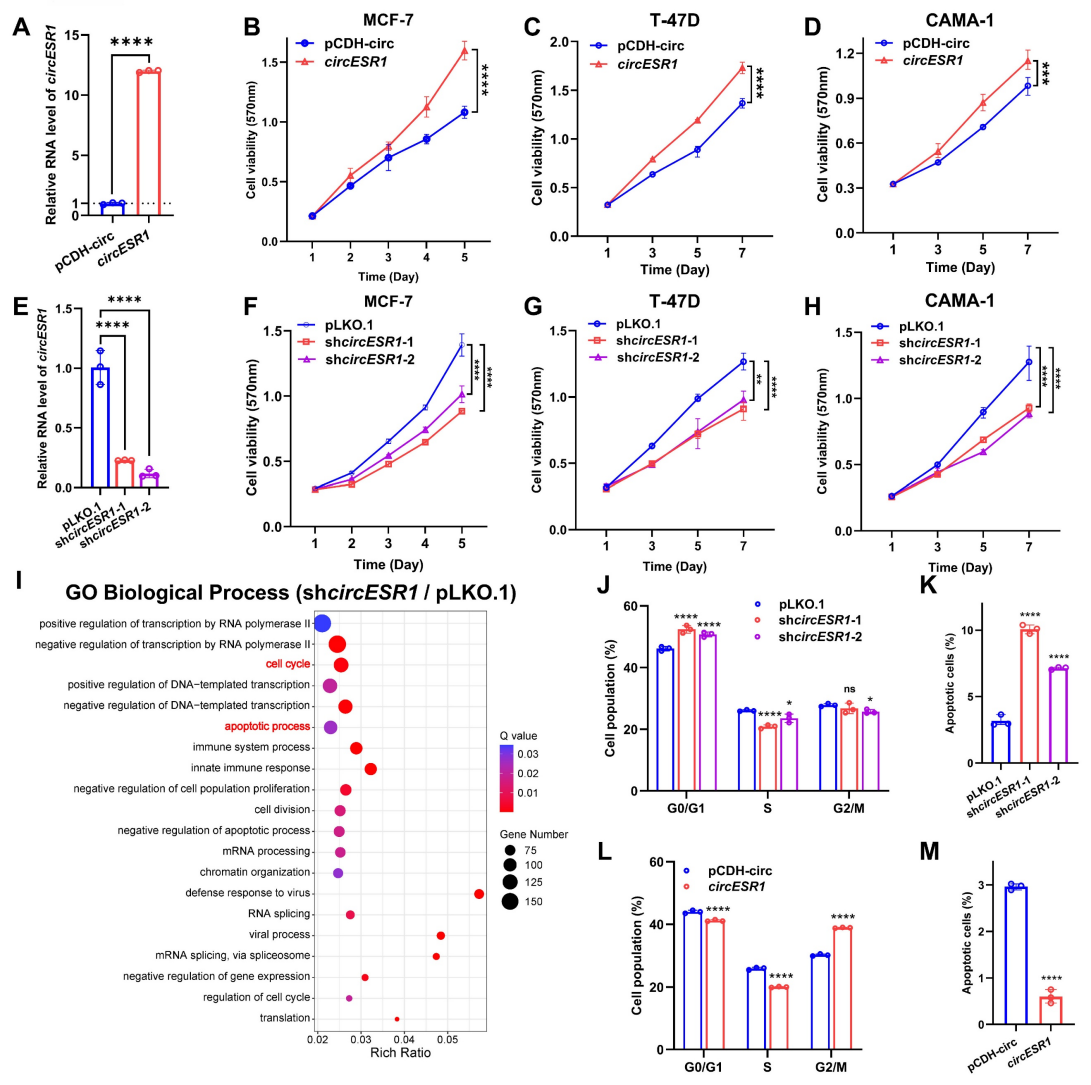
***CircESR1* promotes cell cycle transition of ER+ BC cells.** (A) The relative expression of *circESR1* in MCF-7 cells bearing control vector or vector expressing *circESR1* analyzed by qRT-PCR. (B-D) Cell viability in ER+ BC cells bearing control vector or vector expressing *circESR1* determined by MTT assay. (E) The relative expression of *circESR1* in MCF-7 cells bearing control or *circESR1* shRNAs analyzed by qRT-PCR. (F-H) Cell viability in ER+ BC cells bearing control or *circESR1* shRNAs determined by MTT assay. (I) GO enrichment analysis of biological processes in MCF-7 cells bearing control or *circESR1* shRNAs. *P value* was adjusted using the Benjamini-Hochberg method to obtain *Q value* (FDR-corrected *p-value*). Terms with *Q value* < 0.05 were considered significantly enriched. (J-K) Flow cytometry showed the cell cycle distribution and total proportion of cell apoptosis (Annexin V-FITC+) in MCF-7 cells bearing control or *circESR1* shRNAs. (L-M) Flow cytometry showed the cell cycle distribution and total proportion of cell apoptosis (Annexin V-FITC+) in MCF-7 cells bearing control vector or vector expressing *circESR1*. Data was shown as mean ± S.D. from three independent experiments. Unpaired two-tailed Student's *t* test (A, L-M) and one-way ANOVA followed by Tukey's multiple comparisons test (E, J-K) and two-way ANOVA test (B-D, F-H). ns, *P*>0.05; *, *P*<0.05; **, *P*<0.01; ***, *P*<0.001; ****, *P*<0.0001.

Functionally, forced expression of *circESR1,* but not cognate linear sequences, promoted the viability of ER+ BC cells (MCF-7, T-47D and CAMA-1) shown by using MTT (Figure 2A-D and Figure S4A-C) or foci formation assay (Figure S2A-B), whereas *circESR1* depletion by shRNAs repressed the viability of ER+ BC cells (Figure 2E-H and Figure S2C-D). Unbiased transcriptome sequencing (bulk RNA-seq) was performed to identify differentially expressed genes (DEGs) as a result of *circESR1* depletion in MCF-7 cells (Figure 2I and Figure S3E). GO enrichment analysis of biological processes revealed that *circESR1* was mainly involved in cell cycle and apoptotic process. Consistently, the percentage of G0/G1 phase arrested cells increased while S/G2/M phase decreasing significantly upon *circESR1* silencing in MCF-7, T-47D and CAMA-1 cells, whereas *circESR1* depletion increased the percentage of apoptotic cells (Figure 2J-K and Figure S2F-O). Conversely, forced expression of *circESR1* but not its cognate linear sequence led to a significantly increased cell proportion in S/G2/M phase of MCF-7, T-47D and CAMA-1 cells, and a significantly reduced ratio of cell apoptosis (Figure 2L-M, Figure S3-4). Consistently, *in vivo* studies confirmed that *circESR1* depletion significantly abrogated xenograft growth of MCF-7 cells (Figure 4E-G and Figure S10A-C). Notably, forced expression of *circESR1* in normal breast epithelial MCF-10A and HMEC-hTERT cells, neither of which express ERα, promoted cell viability (Figure S5A-C) and led to a significantly increased cell proportion in S/G2/M phase, suggesting that *circESR1* propelled proliferation and cell cycle transition is independent of ERα activity (Figure S5D-E).

### *CircESR1* interacts with and stabilizes HNRNPAB

**Figure 3:**
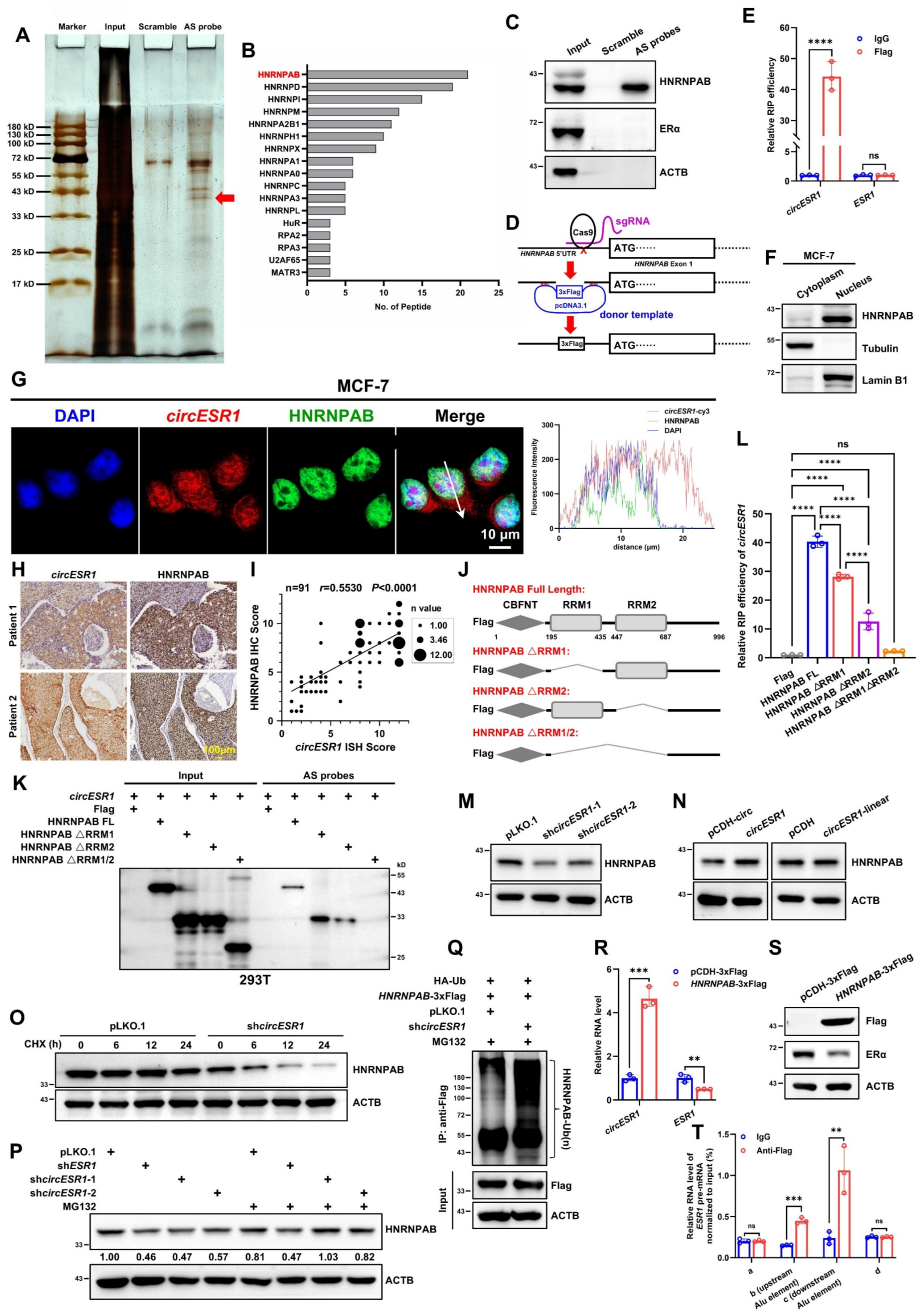
***CircESR1* interacts with HNRNPAB in the biogenesis of *circESR1*.** (A) Potential *circESR1*-associated proteins identified via SDS-PAGE followed by silver staining. The red arrow denotes the band identified as HNRNPAB by MS. (B) Potential *circESR1*-associated proteins in MCF-7 cells analyzed by mass spectrometry and sorted in descending order of peptide number. (C) Immunoblot assessment of HNRNPAB expression in pull-down results, and ERα and ACTB expression were used as negative control. (D) The schematic of inserting 3x Flag tags before the start codon of HNRNPAB protein genome using CRISPR knock-in. (E) RIP and qRT-PCR performed using IgG and anti-Flag antibodies in MCF-7 cells expressing HNRNPAB-3×Flag protein endogenously. (F) Detected the cellular subcellular localization of HNRNPAB after nuclear and cytoplasmic separation of MCF-7 cells. (G) Immunofluorescence analysis localization of DAPI (blue), *circESR1* AS probe labeled with cy3 (red) and anti-HNRNPAB antibody (green) in MCF-7 cells. Scale bars, 10 μm. (H) IHC staining of HNRNPAB protein and ISH staining of *circESR1* in serial tissue sections of ER+ BC patients. Scale bar, 100 μm. (I) According to the ISH staining results of *circESR1* and the IHC staining results of HNRNPAB in serial tissue sections derived from paraffin-embedded ER+ BC specimens, the correlation between *circESR1* and HNRNPAB was quantitatively analyzed according to the average score of each section. The size of each data point represented the statistical n value, with a total of n=91. *P* value was determined by Pearson correlation analysis. (J) Schematic diagram of constructing different truncated mutation vectors for the functional domains of HNRNPAB protein. (K) Immunoblot assessment of RNA pull-down experiments in 293T cells, which transiently transferred *circESR1* and different truncated mutation vectors of HNRNPAB. The results were incubated with Flag antibody for detection. (L) The relative expression of *circESR1* in RIP assay using anti-Flag antibody in MCF-7 cells transiently transferred with different truncated mutation vectors of HNRNPAB analyzed by qRT-PCR. *P* value was determined by Pearson correlation analysis. (M-N) Immunoblot assessment of HNRNPAB expression after changing *circESR1* or cognate linear sequences in MCF-7 cells. (O) Half-life of HNRNPAB transcript was determined in MCF-7 cells transfected with *circESR1* or control and further treated with 100 μg/mL cycloheximide. (P) Immunoblot assessment of HNRNPAB expression with or without MG-132 after knocking down *ESR1* mRNA or *circESR1* in MCF-7 cells. (Q) MCF-7 cells co-transfected with *HNRNPAB*-3xFlag and HA-Ub were immunoprecipitated with anti-Flag antibody after treatment of 20 μmol/L MG132 for 6 hours. Ubiquitinated HNRNPAB-3xFlag was detected by using anti-HA antibody. (R) The relative expression of *circESR1* and *ESR1* mRNA in MCF-7 cells bearing control vector or vector expressing HNRNPAB analyzed by qRT-PCR. (S) Immunoblot assessment of HNRNPAB and ERα expression in (R). (T) The relative expression of Alu sequence on both sides of the upstream and downstream of *circESR1* in *ESR1* pre-mRNA (b and c regions) by qRT-PCR. The amplified template was the sample of MCF-7 cells with endogenous HNRNPAB-3×Flag protein for ultrasonic and CLIP experiments. The regions outside of Alu sequence on both sides (a and d regions) were as negative controls. Data was shown as mean ± S.D. from three independent experiments. Unpaired two-tailed Student's *t* test (E, R) and one-way ANOVA followed by Tukey's multiple comparisons test (L). ns, *P*>0.05; **, *P*<0.01; ***, *P*<0.001; ****, *P*<0.0001.

For mechanistic insight, RNA pull-down of *circESR1* followed by mass spectrometry (MS) analysis was performed (Figure 3A and Table S10). HNRNPAB was identified as a potential putative *circESR1* interacting protein based on peptide abundance in MS (Figure 3B). It was verified by RNA pull-down that HNRNPAB, but not ERα, specifically interacted with *circESR1* (Figure 3C and Figure S6A). CRISPR/Cas9 technology was applied to knock-in a start codon and 3xFlag coding sequences in the 5'UTR of the* HNRNPAB* gene in MCF-7 cells (Figure 3D and Figure S6B). RNA-binding protein immunoprecipitation (RIP) assays subsequently confirmed the enrichment of *circESR1*, but not *ESR1* mRNA, in HNRNPAB complexes precipitated with anti-Flag antibody compared to the control IgG (Figure 3E). Conversely, RIP assays verified that ERα interacted with *GREB1* mRNA rather than *circESR1* in ERα complexes precipitated with anti-Flag antibody compared to the control IgG 19*,* suggesting no direct interaction was detected between ERα and *circESR1* (Figure S6C). Moreover, HNRNPAB was predominantly localized in the nucleus (Figure 3F and Figure S6D) and co-localized with *circESR1* in the nucleus as determined by IF and FISH staining (Figure 3G and Figure S6E). IHC and ISH staining were performed by using serial tissue sections derived from 91 paraffin-embedded ER+ BC specimens. It was observed that the expression level of *circESR1* correlated with that of HNRNPAB in ER+ BC patient samples (Figure 3H). Statistical analysis of IHC and ISH staining score revealed that *circESR1* expression was positively correlated with that of HNRNPAB (*r* = 0.5530; Figure 3I).

The domain of HNRNPAB by which interaction with *circESR1* is mediated was mapped. As HNRNPAB possesses two low complexity regions and two RNA recognition motifs (RRM) based on the SMART database, HNRNPAB mutants with truncated RRM domains were constructed (Figure 3J). HNRNPAB plasmids with different truncations and *circESR1* plasmid were transiently co-transfected into 293T cells, which endogenously expressed neither *ESR1* mRNA nor *circESR1*, for RNA pull-down. It was observed that *circESR1* could interact with wild type HNRNPAB or its cognate mutants with either RRM domain deletion, but not the mutant with both RRM domains deleted (Figure 3K). Reciprocal RIP experiments in MCF-7 or 293T cells confirmed that either of two RRM domains was required for recruiting *circESR1* (Figure 3L and Figure S6F-I). It is fascinating that RRM2 deletion of HNRNPAB resulted in much reduced recruitment of *circESR1* compared to a moderate reduction upon RRM1 deletion, suggesting that *circESR1* preferentially binds to the RRM2 domain of HNRNPAB (Figure 3L and Figure S6F-H). To better understand the formation of HNRNPAB-*circESR1* complex from the perspective of bioinformatics, we uploaded the predicted HNRNPAB tertiary structure from the Alphafold website and the *circESR1* tertiary structure constructed from RNAComposer website to the HDOCK website for binding prediction. Consistent with the results from RNA pull-down and RIP assays, molecular docking analysis suggested that the tertiary structure of *circESR1* possesses a high binding affinity for HNRNPAB, whereas single deletion of either RRM domain cannot abolish the theoretical capacity of *circESR1* to bind to HNRNPAB (Figure S6I).

Whether HNRNPAB levels were affected by *circESR1* interaction was also examined in MCF-7 cells. Interestingly, *circESR1* depletion reduced HNRNPAB expression, whereas forced expression of *circESR1* but not cognate linear sequences promoted HNRNPAB expression (Figure 3M-N). The possible influence on HNRNPAB protein stability was further determined in presence of cycloheximide (CHX) treatment. Consistently, *circESR1* depletion reduced the half-life of HNRNPAB (Figure 3O and Figure S6J) and MG132 treatment restored *circESR1* depletion decreased HNRNPAB expression (Figure 3P). An increased ubiquitination level of HNRNPAB due to *circESR1* depletion was also observed compared to the control (Figure 3Q). Thus, *circESR1* preferentially interacts with the RRM2 domain of HNRNPAB to increase its stability and expression via the ubiquitin-proteasome proteolytic pathway.

### HNRNPAB promotes the biogenesis of *circESR1*

Whether HNRNPAB could affect *circESR1* expression in return was next investigated. It was observed that forced expression of HNRNPAB increased* circESR1* expression and concomitantly reduced the expression of *ESR1* mRNA and ERα (Figure 3R-S). We further determined the possible regulation of *circESR1* biogenesis by HNRNPAB. At present, two predominant mechanisms for the generation of circRNAs from the host pre-mRNAs have been documented 1, 45, 52, including duplication elements (such as Alu sequences) mediating the complementary pairing of the wing sequence of circRNAs, and the wing sequence of circRNAs combined with RNA binding protein (RBP) to form a dimer. By using the UCSC Genome Browser, the nearest Alu sequences on both sides of *circESR1* were located (Figure S6K). PCR amplification primers based on the predicted Alu sequence (b and c regions in Figure S6L) and the regions outside of Alu sequence on both sides as negative controls were designed (a and d regions in Figure S6L). Crosslinking-immunprecipitation (CLIP) assay using the anti-Flag antibody followed by qRT-PCR revealed that HNRNPAB bound to the Alu sequences on both sides of *circESR1* compared to IgG group. The regions outside of the Alu sequence on both sides were selected as negative control for qRT-PCR detection and showed that HNRNPAB did not bind to these regions (Figure S6L and Figure 3T). It was further shown that only wild type HNRNPAB, but not its mutants with either RRM domain truncations, promoted the biogenesis of *circESR1* (Figure S6M). It is apparent that HNRNPAB promotes the biogenesis of *circESR1* by combining the two Alu sequences of *ESR1* pre-mRNA to boost circRNA cyclization.

### HNRNPAB mediates *circESR1* promoted cell cycle transition

**Figure 4:**
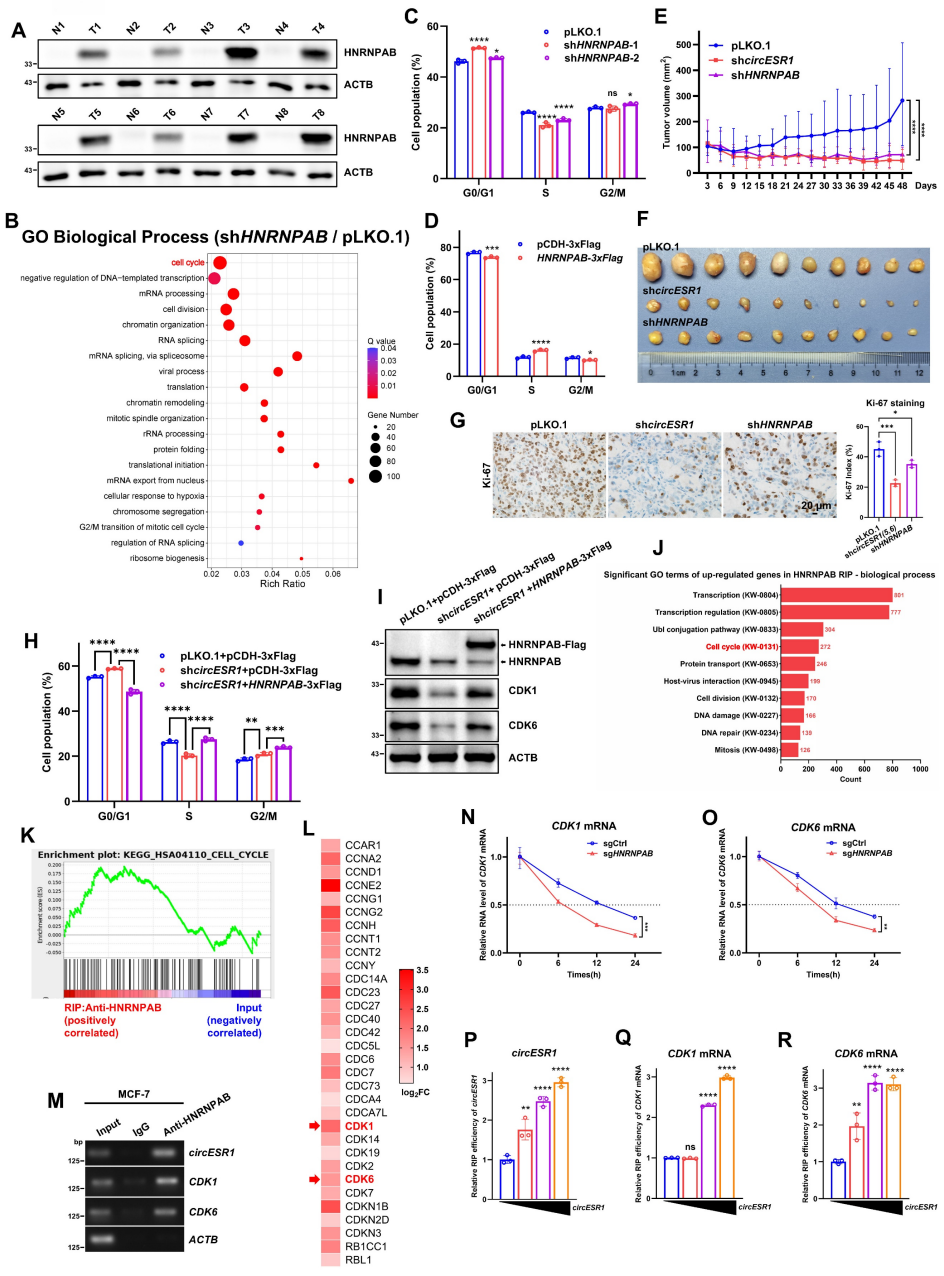
** HNRNPAB mediates *circESR1* promoted cell cycle transition.** (A) Immunoblot assessment of HNRNPAB in adjacent (N) and cancerous (T) tissues of ER+ BC patients. (B) GO enrichment analysis of biological processes in MCF-7 cells bearing control or *HNRNPAB* shRNAs.* P value* was adjusted using the Benjamini-Hochberg method to obtain *Q value* (FDR-corrected *p-value*). Terms with *Q value* < 0.05 were considered significantly enriched. (C) Flow cytometry showed the cell cycle distribution in MCF-7 cells bearing control or *HNRNPAB* shRNAs. (D) Flow cytometry showed the cell cycle distribution in MCF-7 cells bearing control vector or vector expressing *HNRNPAB*. (E-F) Changes in tumor growth volume in xenograft mouse models. Injected with 2×10^6^ MCF-7 control or knockdown *circESR1* or knockdown *HNRNPAB* cells under the second pair of fat pads on both sides of the mammary glands of female BALB/c nude mice (n=5/group). (G) IHC for Ki-67 in tumor sections derived from (F). Scale bars, 20 μm. The staining of Ki-67 in tumor sections was assessed by H-score (Right). (H) Flow cytometry showed the cell cycle distribution in MCF-7 cells with or without *circESR1* shRNAs in the presence or absence of overexpressing HNRNPAB. (I) Immunoblot assessed the expression of cell cycle related genes in (H). (J) GO analysis of the biological process of RIP-seq enriched RNA by anti-HNRNPAB antibody. (K) GSEA enrichment analysis of the correlation between anti-HNRNPAB antibody enrichment group and cell cycle gene set. (L) Selected the RNAs with a relative expression level 1.5 times higher than that of the Input group in anti-HNRNPAB antibody enrichment group from 272 cell cycle related RNAs, and display the log_2_FC values in a heatmap. (M) The mRNAs enriched by HNRNPAB in RIP-seq was verified in MCF-7 cells, and was displayed by agarose gel electrophoresis after analyzed by qRT-PCR. (N-O) Treated MCF-7 control or knockdown HNRNPAB cells with gradient time of actinomycin D (0, 6, 12, 24 h) and detected the degradation rate of *CDK1* and *CDK6* mRNAs. (P-R) 293T cells co-transfected with the full-length of HNRNPAB (15 μg) and gradient amounts of *circESR1* (1, 5, 9, 13 μg) were used for RIP assay by use of anti-Flag antibody, then the relative expression of *CDK1* and *CDK6* mRNAs analyzed by qRT-PCR. Data was shown as mean ± S.D. from three independent experiments. Unpaired two-tailed Student's *t* test (D) and one-way ANOVA followed by Tukey's multiple comparisons test (C, G-H, P-R) and two-way ANOVA test (E, N-O). ns, *P* > 0.05; *, *P* < 0.05; **, *P* < 0.01; ***, *P* < 0.001; ****, *P* < 0.0001.

Analysis of HNRNPAB expression by using the GEPIA2 tool suggested that *HNRNPAB* mRNA levels were markedly increased in BC tissues compared with normal tissues (Figure S7A). Elevated levels of HNRNPAB were observed in primary BC tissues by analyzing the BC dataset of Clinical Proteomic Tumor Analysis Consortium (CPTAC) (Figure S7B). By using tissues collected from BC patients, an increased HNRNPAB expression in ER+ BC tissues compared to adjacent non-tumor tissues was also observed (Figure 4A). Further correlation of HNRNPAB expression with clinicopathological characteristics of ER+ BC patients revealed a strong correlation with high ERα expression, increased lymph node metastasis and Ki-67 positivity (Table 2 and Figure S8B). KM Plotter analysis also showed that ER+ BC patients with higher tumor expression of HNRNPAB exhibited worse overall survival and disease-free survival outcomes (Figure S7C-D). Unbiased transcriptome sequencing (bulk RNA-seq) was performed to identify differentially expressed genes (DEGs) regulated by HNRNPAB. GO enrichment analysis of biological processes revealed that HNRNPAB was mainly involved in cell cycle and apoptotic processes (Figure 4B and Figure S7E). Functional analysis of HNRNPAB at the single-cell level from CancerSEA website suggested a similar role of HNRNPAB in cell cycle progression of BC (Figure S7F-G).

Consistent with clinical data and RNA-seq analysis, HNRNPAB depletion led to decreased foci formation (Figure S9A) and increased the percentage of apoptotic cells (Figure S9F-H and Figure S9K-M). The percentage of G0/G1 phase arrested cells increased whereas S/G2/M phase cells decreased significantly upon HNRNPAB silencing (Figure 4C, Figure S9C-E and Figure S9I-J). In contrast, forced expression of HNRNPAB led to increased foci formation, increased cells in the S/G2/M phase and reduced cell apoptosis (Figure 4D, Figure S9B and Figure S9N-O). Consistently, *in vivo* studies confirmed that HNRNPAB depletion significantly abrogated ER+ BC xenograft growth (Figure 4E-G and Figure S10A-C). It was further observed that HNRNPAB forced expression restored *circESR1* depletion rendered cell cycle arrest and reduced expression of cell cycle related proteins (Figure 4H-I and Figure S10D-F), suggesting that HNRNPAB mediated *circESR1* promoted cell cycle transition.

### HNRNPAB promotes cell cycle transition by interacting with *CDK1* and *CDK6* mRNA

To determine if HNRNPAB could interact with mRNAs, RIP-seq was performed by using an anti-HNRNPAB antibody with *circESR1* enrichment monitored (Figure 4M and Figure S10H). GO enrichment analysis combined with GSEA revealed the enrichment of mature mRNAs of cell cycle-related genes by HNRNPAB compared to those with input (Figure 4J-K). Comparison of RIP-seq data identified 32 cell cycle related mRNAs with contrast changes from the HNRNPAB enriched group compared to those from the input (fold change > 1.5, Figure 4L). Among those, *CDK1* and *CDK6* mRNAs were subsequently verified to interact with HNRNPAB (Figure 4M and Figure S10G). Although the interaction of *CDK4* mRNA with HNRNPAB was reported in lung adenocarcinoma 14, we failed to identify *CDK4* in both the RIP-seq data and verification of RIP experiments by using anti-HNRNPAB antibody. HNRNPAB depletion reduced the half-life of *CDK1* and *CDK6* mRNAs (Figure 4N-O), suggesting a role of HNRNPAB in stabilizing these RNA transcripts. Consistent with this role in mRNA stabilization, depletion of either *circESR1* or HNRNPAB reduced CDK1 and CDK6 protein levels (Figure S10H-I). Of note, HNRNPAB expression strongly correlated with CDK1 (*r* = 0.5456) and CDK4 (*r* = 0.3212) levels in 68 cases of ER+ BC by analyzing the BC dataset of CPTAC (Figure S10J-K).

RIP analysis revealed that *CDK1* and *CDK6* mRNAs interacted with wild type HNRNPAB or HNRNPAB with RRM2 domain deletion, but not the mutants with RRM1 domain deletion or deletion of both RRM domains, suggesting that the RRM1 domain was exclusively required for mRNA recruitment (Figure S10L-M). To further determine whether the interaction of *circESR1* with HNRNPAB could affect the capacity of HNRNPAB to recruit mRNAs, HNRNPAB plasmid and graded amounts of *circESR1* plasmid were transiently co-transfected into 293T cells for RIP analysis. The data consistently showed that *circESR1* promoted the binding of *CDK1* and* CDK6* mRNAs to HNRNPAB (Figure 4P-R), whereas transfection of increasing amounts of *circESR1* upon RRM2 domain deletion reduced the binding capacity of these mRNAs to HNRNPAB (Figure S10N-P). Thus, *circESR1* predominantly interacted with the RRM2 domain of HNRNPAB to promote the recruitment of *CDK1* and* CDK6* mRNAs. Taken together, HNRNPAB promoted cell cycle transition by interacting with and stabilizing *CDK1* and *CDK6* mRNAs, which was facilitated by the interaction with *circESR1*.

To further understand the binding conformation among HNRNPAB, *circESR1* and cell cycle mRNAs, it was sought to decipher their structural basis by a bioinformatics approach. As the RNAComposer could only predict the tertiary structure of RNA with no more than 500 residues, we first used the catRAPID website to predict the possible binding position of HNRNPAB protein sequence to either *CDK1* mRNA or *CDK6* mRNA. The predicted results suggested that HNRNPAB may bind to the first 500 residues of the 5' end of the two mRNA sequences (including the 5' UTR region) (Figure S11A-B). RNAComposer was then used to construct the tertiary structure of the first 500 residues of *CDK1* and *CDK6* mRNAs. The conformation of the HNRNPAB-*circESR1* complex (Figure S11I) and the tertiary structure of each mRNA was uploaded to predict their potential binding on the HDOCK website. A possibility of binding between HNRNPAB and each cell cycle mRNA was indeed suggested (Figure S11C-D). To gain insight on the asymmetrical binding of *circESR1* and mRNAs to HNRNPAB, the similarity of two RRM domains of HNRNPAB based on SMART database was also compared with 69% shared similarity in protein sequence observed (Figure S11E). Taken together, HNRNPAB promoted cell cycle transition by interacting with and stabilizing *CDK1* and *CDK6* mRNAs, which was facilitated by the interaction with *circESR1*.

### Estrogen promotes HNRNPAB expression via SP1

**Figure 5:**
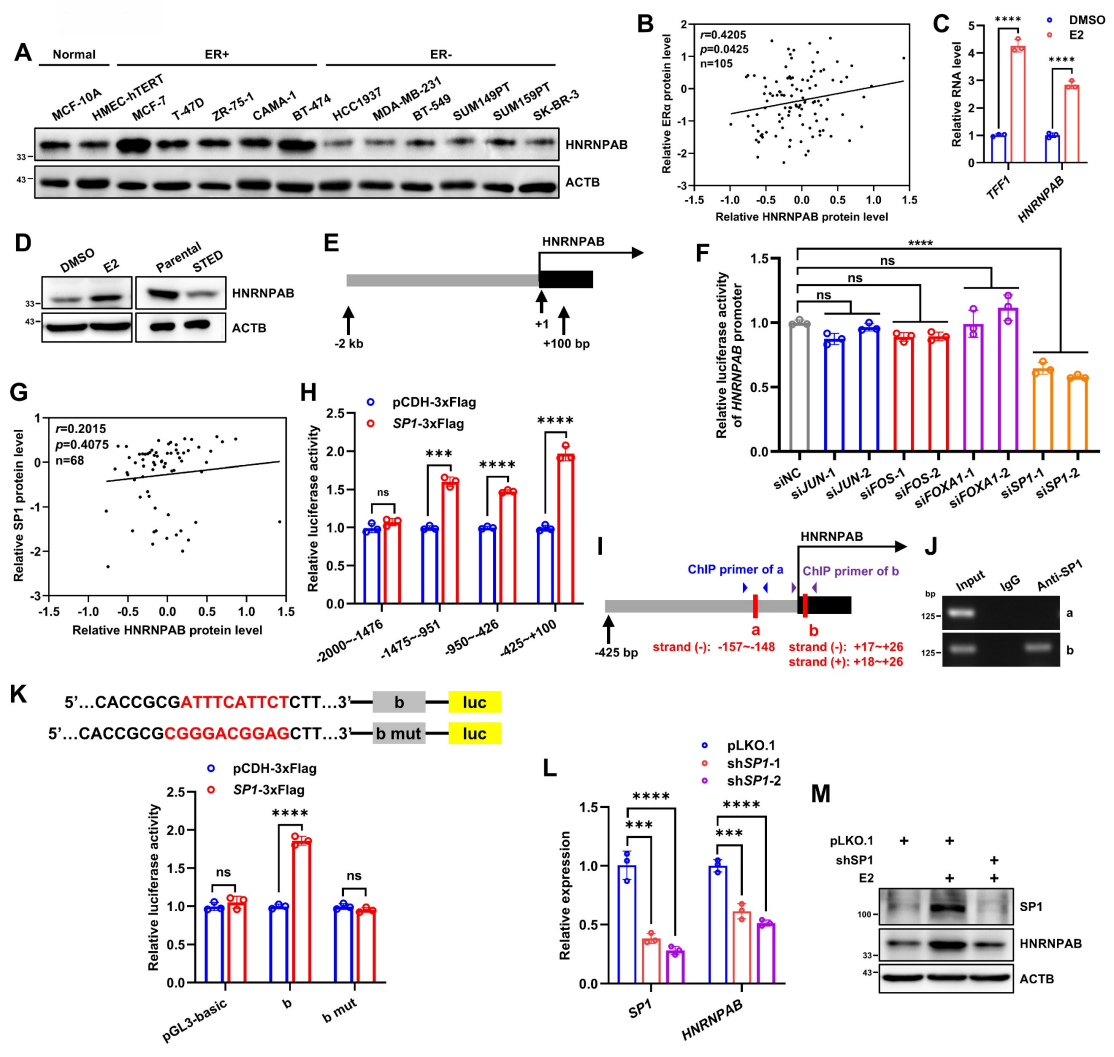
** Estrogen promotes HNRNPAB expression via SP1.** (A) Immunoblot assessment of HNRNPAB in 2 human normal breast epithelial cell lines and 11 human BC cell lines. (B) CPTAC database analyzed the relative HNRNPAB and ERα protein level in 105 BC patients. *P* value was determined by Pearson correlation analysis. (C) The relative expression of *TFF1* and *HNRNPAB* mRNAs after stimulated with 10 nM E_2_ in estrogen-deprived MCF-7 cells for 48 h analyzed by qRT-PCR. (D) Immunoblot assessment of HNRNPAB expression in MCF-7 with or without 10 nM E_2_ in estrogen-deprived MCF-7 cells for 72 h, or in short-term oestrogen deprivation (STED) MCF-7 cells for 7 days and parental cells. (E) The schematic of *HNRNPAB* promoter region sequence selected from the transcription start site of -2 kb~+100 bp. (F) MCF-7 cells were transfected with pGL3 basic reporter vector containing HNRNPAB promoter, and pRL-TK reporter control vector containing luciferase activity, as well as control siRNA or different siRNA sequences of *JUN*, *FOS*, *FOXA1* and *SP1*. The relative fluorescence activity was measured. (G) CPTAC database analyzed the relative HNRNPAB and SP1 protein level in 68 ER+ BC patients. *P* value was determined by Pearson correlation analysis. (H) 293T cells were transfected with control or SP1 vector, and pRL-TK reporter control vector containing luciferase activity, as well as pGL3 basic reporter vector containing different regions of HNRNPAB promoter. The relative fluorescence activity was measured. (I) The schematic of three predicted conserved SP1 binding sites on the upstream -425 bp~+100 bp regions of the human* HNRNPAB* gene. Red boxes represent the predicted SP1 binding sites. (J) The DNA regions enriched by SP1 in ChIP assay were verified by PCR. (K) 293T cells were transfected with control or SP1 vector, and pRL-TK reporter control vector containing luciferase activity, as well as pGL3 basic reporter vector or containing “b” or “b” mutant regions. The relative fluorescence activity was measured. (L) The relative expression of *SP1* and *HNRNPAB* mRNAs in MCF-7 cells bearing control or *SP1* shRNAs analyzed by qRT-PCR. (M) Immunoblot assessment of SP1 and HNRNPAB expression in MCF-7 cells with or without *SP1* shRNAs in the presence or absence of 10 nM E_2_ for 48 h. Data was shown as mean ± S.D. from three independent experiments. Unpaired two-tailed Student's *t* test (C, H, K) and one-way ANOVA followed by Tukey's multiple comparisons test (F, L). ns, *P*>0.05; ***, *P*<0.001; ****, *P*<0.0001.

It is interesting to note that MG132 treatment did not restore *ESR1* depletion decreased HNRNPAB expression (Figure 3P), implying that alternative mechanisms might be employed to regulate HNRNPAB expression. In this regard, our attention was attracted by the interesting association between HNRNPAB and ERα expression. Elevated expression of HNRNPAB was observed in five ER+ BC cells compared to two normal breast epithelial and six ER- BC cells (Figure 5A). Consistently, HNRNPAB protein level was strongly correlated with ERα protein expression in 105 BC cases by analyzing the BC dataset of CPTAC (*r* = 0.4205, Figure 5B). Estrogen treatment significantly increased HNRNPAB expression at both mRNA and protein levels (Figure 5C-D), whereas estrogen deprivation and fulvestrant treatment remarkably decreased HNRNPAB expression (Figure 5D and Figure S14C-D), suggesting a dependence on ER signaling for HNRNPAB expression.

To determine the mechanism of estrogen regulated HNRNPAB expression, the binding sites of ER or other possible estrogen-related transcription factors on the -2 kb~+100 bp promoter regions upstream of *HNRNPAB* gene were predicted using tools from JASPAR and rVista 2.0 53, 54, 55, 56. Although no canonical estrogen response element (ERE) sites were predicted, multiple binding sites of AP1 (JUN and FOS), FOXA1 and SP1 were identified. The promoter DNA upstream of the *HNRNPAB* gene was cloned into a luciferase reporter vector (Figure 5E). With shRNA efficiency confirmed (Figure S12A-D), the depletion of SP1, but not JUN, FOS or FOXA1, reduced the luciferase activity from the transfected reporter plasmid, suggesting the transcriptional activation of *HNRNPAB* by SP1 (Figure 5F). Indeed, 119 and 20 SP1 binding sites on the *HNRNPAB* promoter were predicted by JASPAR and rVista 2.0, respectively. SP1 expression was also positively correlated with HNRNPAB levels in 68 cases of ER+ BC by analyzing the BC dataset of CPTAC (Figure 5G). To define the mechanism of transcriptional regulation of HNRNPAB by SP1, we subdivided the first 2 kb of the HNRNPAB promoter region into four ~500 bp segments and further subcloned them into the luciferase reporter plasmid. It was observed that SP1 transfection led to maximal transcriptional activity of a luciferase reporter containing the -425 bp~+100 bp promoter regions compared to other three reporter plasmids (Figure 5H). Three predicted conserved SP1 binding sites in the -425 bp~+100 bp regions upstream of the human and house mouse* HNRNPAB* gene were located using the JASPAR database (Figure 5I). Chromatin immunoprecipitation (ChIP) was performed in MCF-7 cells using SP1 antibody. Given that two predicted SP1 binding sites were in the same DNA regions of two strands, we designed primers for “a” and “b” regions and verified that SP1 bound to the chromatin fragment comprising of the b-site (Figure 5J). Consistently, mutation of the b-site abrogated the increased reporter activity observed with forced expression of SP1 (Figure 5K).

As expected, SP1 depletion reduced *HNRNPAB* mRNA expression (Figure 5L). We showed further that estrogen treatment potently increased the expression of SP1 and HNRNPAB, whereas SP1 depletion abrogated estrogen increased HNRNPAB expression (Figure 5M). Consistently, fulvestrant treatment remarkably reduced the expression of SP1 and HNRNPAB in ER+ BC cells (Figure S14C-D). Thus, estrogen promotes HNRNPAB expression through SP1.

### *CircESR1* promotes antiestrogen resistance via HNRNPAB

**Figure 6:**
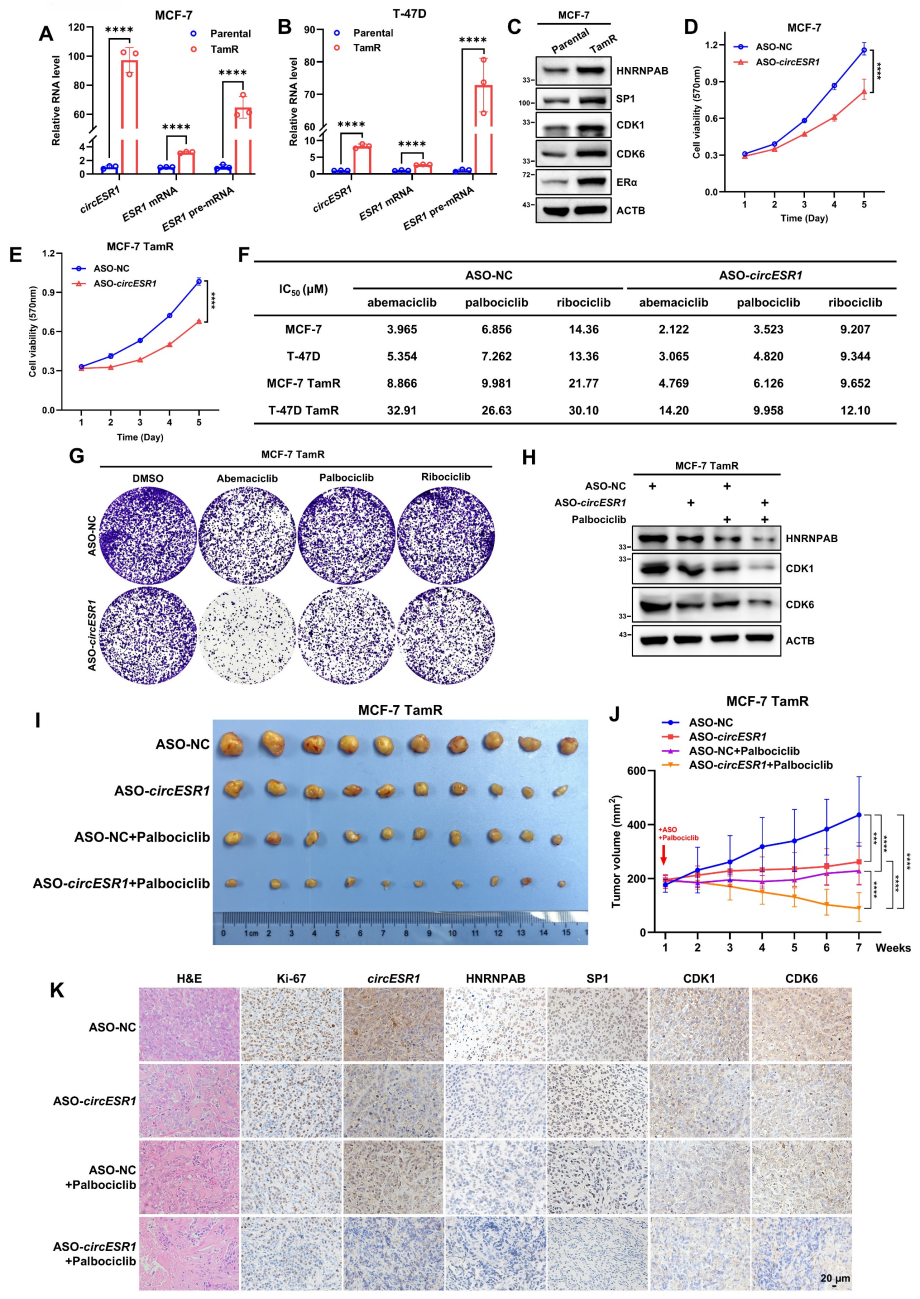
** Combined treatment of antiestrogen-resistant ER+ BC with ASO targeting *circESR1* and CDK4/6i.** (A-B) The relative expression of *circESR1*, *ESR1* mRNA and *ESR1* pre-mRNA in MCF-7 or T-47D parental and tamoxifen resistant (TamR) cells analyzed by qRT-PCR. (C) Immunoblot assessed the expression of HNRNPAB, SP1, CDK1, CDK6 and ERα proteins in MCF-7 parental and TamR cells. (D-E) Cell viability in MCF-7 parental and TamR cells bearing control ASO or ASO targeting *circESR1* determined by MTT assay. (F) MCF-7 and T-47D parental and TamR cells were transiently transfected with control ASO or ASO targeting *circESR1*, and were treated with different concentration gradients of CDK4/6i for 48 h. The drug killing curve of the cells was detected by MTT assay. (G) Foci formation to detect the effects of combining abemaciclib, palbociclib, and ribociclib on MCF-7 TamR cells transiently transfected with control ASO or ASO targeting *circESR1*. (H) Immunoblot assessment of HNRNPAB, CDK1 and CDK6 proteins in MCF-7 TamR cells, which were transiently transfected with control ASO or ASO targeting *circESR1* and treated with palbociclib for 48 h. (I-J) Changes in tumor growth volume in xenograft mouse models. Injected with 1×10^6^ MCF-7 TamR cells under the second pair of fat pads on both sides of the mammary glands of female BALB/c nude mice (n=5/group). Tamoxifen (20 μg per dose) dissolved in 125 μL corn oil was injected every 3 days i.p. When the xenograft volume reached approximately 200 mm^3^, tumor-bearing mice were randomized and received intratumoral injection of negative control or ASO-*circESR1* (5nM per dose, every 3 days) in the presence or absence of palbociclib (100mg/kg/week i.g.). (K) Hematoxylin and eosin (H&E) staining, ISH for *circESR1* and IHC for Ki-67, HNRNPAB, SP1, CDK1 and CDK6 in tumor sections derived from (I). Scale bars, 20 μm. Data was shown as mean ± S.D. from three independent experiments. Unpaired two-tailed Student's *t* test (A-B) and two-way ANOVA test (D-E, J). ***, *P* < 0.001; ****, *P* < 0.0001.

As ER signaling is intricately associated with antiestrogen therapy of BC, the possible involvement of *circESR1* and HNRNPAB in antiestrogen sensitivity was determined. *CircESR1* forced expression in MCF-7 cell reduced cell sensitivity to tamoxifen (Figure S13A). Acquired antiestrogen resistance models, MCF-7 tamoxifen resistant (TamR) and T-47D TamR, were also utilized with prior verification (Figure S13C-D). Interestingly, a significantly higher level of *circESR1*, *ESR1* mRNA and *ESR1* pre-mRNA was observed in TamR cells compared to the parental cells (Figure 6A-B). Functionally, forced expression of *circESR1* but not the cognate linear sequences increased foci formation of MCF-7 TamR cells (Figure S13E), whereas c*ircESR1* depletion reduced foci formation, promoted G0/G1 phase arrest and restored the sensitivity of MCF-7 TamR cells to tamoxifen (Figure S13G-J). As expected, *circESR1* depletion reduced HNRNPAB levels in TamR cells (Figure S13F and Q).

Similarly, forced expression of HNRNPAB reduced the sensitivity to tamoxifen in MCF-7 cells (Figure S13B). Increased expression of HNRNPAB was observed in TamR cells compared to the parental cells (Figure 6C and Figure S13K). Furthermore, increased expression of SP1, CDK1, CDK6 and ERα were observed in MCF-7 TamR cells (Figure 6C). Consistent with their dependence on ERα signaling, fulvestrant treatment decreased SP1 and HNRNPAB expression in MCF-7 TamR cells (Figure S13L). Functionally, HNRNPAB depletion reduced foci formation, promoted G0/G1 phase arrest and restored the sensitivity to tamoxifen of MCF-7 TamR cells (Figure S13M-P). Consistently, *circESR1* or HNRNPAB depletion reduced HNRNPAB, CDK1 and CDK6 protein levels in MCF-7 TamR cells (Figure S13Q-R). It was further shown that increased tamoxifen resistance upon forced expression of *circESR1* was substantially mitigated by HNRNPAB depletion (Figure S13S), indicating that *circESR1* promoted antiestrogen resistance of MCF-7 TamR cells via HNRNPAB.

### Combined treatment of antiestrogen-resistant ER+ BC with ASO targeting *circESR1* and CDK4/6i

CDK4/6 inhibitors (CDK4/6is) are among the first or second lines of treatment for hormone receptor-positive/HER2-negative BC, usually combined with antiestrogen therapy 57. Whether CDK4/6is could impinge on the expression of *circESR1* or HNRNPAB was examined by treating ER+ BC cells with three different CDK4/6 inhibitors for 48 hr. Interestingly, reduced expression of HNRNPAB at both mRNA and protein levels was observed in all CDK4/6i treated groups (Figure S14A-B), whereas abemaciclib or ribociclib treatment only slightly increased the expression of *circESR1*. *circESR1* levels remained unaltered as a result of palbociclib treatment (Figure S14A-B). Consistently, reduced expression of SP1, CDK1, ERα, GREB1 and TFF1 were observed after treatment with fulvestrant or different CDK4/6 inhibitors (Figure S14C-D). Whether *circESR1* and/or HNRNPAB regulated the sensitivity to CDK4/6i treatment was further examined. Interestingly, depletion of c*ircESR1* or HNRNPAB increased the efficacy of CDK4/6i treatment in both parental and TamR cells (Figure S14E-F). It was further shown that increased sensitivity to CDK4/6i upon *circESR1* depletion was abrogated by forced expression of HNRNPAB in parental and TamR cells (Figure S14E-F). Hence, *circESR1* depletion enhanced the sensitivity of ER+ BC cells to CDK4/6i via HNRNPAB.

The possibility of targeting *circESR1* in breast cancer therapy was further explored. Antisense oligonucleotides (ASO) were constructed to target *circESR1* for degradation. The efficacy of ASO targeting *circESR1* was determined (Figure S14G). Transient transfection of ASO-*circESR1* reduced the proliferation of parental and TamR cells (Figure 6D-E). ASO targeting *circESR1* also restored the efficacy of tamoxifen in MCF-7 TamR cells (Figure S14H).

Notably, *circESR1* antagonism by ASO increased the efficacy of CDK4/6i treatment as evidenced by a reduced IC_50_ in both parental and TamR cells (Figure 6F). ASO targeting *circESR1* significantly reduced foci formation of MCF-7 TamR cells (Figure 6G) treated with CDK4/6i. It was further shown that ASO targeting *circESR1* combined with palbociclib treatment significantly reduced the expression of HNRNPAB, CDK1 and CDK6 protein in MCF-7 TamR cells (Figure 6H).

Next, 1×10^6^ MCF-7 TamR cells were orthotopically injected into the fat pad of female BALB/C nude mice. When xenograft size reached approximately 200 mm^3^, mice were randomized and received intratumoral injection of negative control or ASO-*circESR1* (5nM per dose, every 3 days) in the presence or absence of palbociclib (100 mg/kg/week i.g.). After seven weeks of treatment, xenografts derived from TamR cells were significantly diminished by using ASO targeting *circESR1* or palbociclib alone compared to the control group. Further escalated suppression of xenograft growth in the group of combined therapy with ASO targeting *circESR1* and palbociclib was observed (Figure 6I-J and Figure S14I-J). Xenograft weight was measured in all groups and a synergistic effect of combined therapy with ASO targeting *circESR1* and palbociclib was observed (Figure 6I-J and Figure S14K). H&E staining revealed that xenografts treated with ASO targeting *circESR1* or palbociclib exhibited remarkably decreased tumor cell density and increased fibrosis compared to control (Figure 6K). Furthermore, ISH and IHC analysis showed that tumors derived from TamR xenografts treated with combined therapy of ASO targeting *circESR1* and palbociclib, exhibited the lowest expression levels of Ki-67, *circESR1*, HNRNPAB, SP1, CDK1 and CDK6 among all groups (Figure 6K). These data suggested that the ASO targeting *circESR1* synergized with CDK4/6i in treating tamoxifen-resistant ER+ BC cells by impinging on a series of its downstream signaling molecules.

## Discussion

Even with antiestrogens and/or CDK4/6 inhibitors as the first or second line of treatment for ER+ breast cancer, disease progression, and relapse often inevitably occur. Thus, it is imperative to exploit novel molecular oncogenic drivers other than ERα to improve the current therapeutic regime. In this study, we systematically screened a matrix of host genes involved in ER signaling and subsequently identified *circESR1* as a novel circRNA generated by back-splicing of the *ESR1* gene with highly specific expression in ER+ breast cancer. Further studies revealed that the interplay between *circESR1* and HNRNPAB plays a key role in ER signaling and antiestrogen therapy and also highlights the protential of targeting *circESR1* for cancer therapy.

As a novel transcript from the *ESR1* gene, the key lineage-specific breast cancer oncogene, the abundance of *circESR1* was susceptible to the factors or/and regulatory mechanisms intricately associated with ER signaling. Firstly, *circESR1* was produced from the back-splicing of *ESR1* pre-mRNA. Whereas the activation or antagonism of ER signaling negatively or positively regulated *ESR1* pre-mRNA levels, the expression level of *circESR1* always followed the alteration of *ESR1* pre-mRNA levels in this context, suggesting the level of *ESR1* pre-mRNA largely determined the basal level of *circESR1*. Secondly, as both *ESR1* mRNA and *circESR1* were generated from the canonical splicing or back-splicing of *ESR1* pre-mRNA transcript, there was inevitably an interplay or competition between these two RNA transcripts. Similar competitive situations were reported in the biogenesis of *circMbl*,* cia-cGAS* and *circHuR* 46, 58, 59. Thirdly, HNRNPAB was recruited to promote the back-splicing and expression of *circESR1* by tethering the Alu elements of the *ESR1* pre-mRNA. Interestingly, HNRNPAB was also identified as a novel estrogen responsive gene, the expression of which was transcriptionally activated by estrogen regulated SP1. Apparently, the expression of *circESR1* was subjected to regulation by multiple ER signaling molecules at different levels. Regulating *circESR1* biogenesis in such a sophisticated manner further suggested its critical role in ER signaling and antiestrogen resistance.

Only a few trans-acting factors have been reported to regulate circRNA formation, including QKI 60, MBL 46, and HNRNPL 61. This study added HNRNPAB to this list and showed that HNRNPAB promotes the back-splicing of the* ESR1* gene, leading to enhanced production of *circESR1*. In return, *circESR1* promoted the stability and expression of HNRNPAB by reducing its ubiquitination. Thus, ER signaling regulates the expression of *circESR1* by influencing both the production of *ESR1* pre-mRNA and its back-splicing via HNRNPAB. However, ER signaling exerted limited influence on *circESR1* expression compared to its effect on *ESR1* mRNA expression in ER+ BC cells, which could be explained by the trade-off between estrogen diminished transcription of *ESR1* pre-mRNA 62 and HNRNPAB promoted back-splicing of *circESR1,* which oppositely regulated the abundance of *circESR1*. In comparison, a substantially elevated expression of *circESR1* was observed in TamR cells compared to parental cells. This could be due to simultaneously elevated expression of *ESR1* pre-mRNA and HNRNPAB in TamR cells, which triggered a positive feedback loop between *circESR1* and HNRNPAB to ensure increased expression of both molecules upon the development of antiestrogen resistance.

It was observed herein that *circESR1* promoted both breast cancer cell cycle transition and antiestrogen resistance by forming a functional complex with HNRNPAB. HNRNPAB further recruited and stabilized multiple cell cycle related mRNAs, including *CDK1*, and *CDK6*. Interestingly, the two RRM domains of HNRNAPB interacted with *circESR1* and mRNAs asymmetrically, wherein* circESR1* preferentially bound to the RRM2 domain and RRM1 domain was exclusively required for mRNA recruitment. This observation suggested that *circESR1* was recruited to the RRM2 domain to facilitate the binding of these mRNA transcripts to the RRM1 domain of HNRNPAB. It is of note that deletion of the RRM2 domain still permitted the binding of *circESR1* to the RRM1 domain of HNRNPAB. Transfection of increasing amounts of *circESR1* progressively reduced the recruitment of mRNAs to the RRM1 domain, suggesting certain competition might exist between these two types of RNA transcripts during their binding to the RRM1 domain of HNRNPAB. The reason why *circESR1* preferentially bound to the RRM2 domain could also be partially explained by the possible competition rendered by the mRNAs recruited to the RRM1 domain of HNRNPAB. The exact structural basis for the asymmetrical binding of the two types of RNA transcripts to HNRNPAB remains to be explored. However, it is of note, that due to the technical difficulty, definitive structural analysis of circRNAs has not yet been reported 63. Whereas our biochemical and functional data support a model of domain-specific interactions, direct structural validation, such as *in vitro* reconstitution and high-resolution structural determination of the HNRNPAB-*circESR1* complex will be essential in future studies to precisely map the binding interfaces. This study presented a novel paradigm based on the dynamic interplay of *circESR1* and HNRNPAB, which facilitated circRNA biogenesis and binding of cell cycle related mRNAs for propelling downstream signaling leading to cell cycle progression. *CircESR1* impinges on multiple pro-oncogenic pathways including pro-proliferative and anti-apoptotic signaling. *CircESR1* should therefore be expected to exert its functional roles by interacting with a series of downstream molecules, in addition to HNRNPAB, as suggested by MS analysis of *circESR1* interacting proteins (Figure 3B). It is highly likely that *circESR1* may employ additional signaling molecules to promote cancer cell cycle progression.

The importance of *circESR1* and HNRNPAB in ER+ breast cancer was further reinforced by their high specificity of expression in ER+ BC tissue and a high correlation with ER positivity. Patients with high levels of *circESR1* and HNRNPAB exhibited an advanced prognostic stage and poor survival outcome. Thus, it would be very interesting to further develop *circESR1* and HNRNPAB as highly specific biomarkers for BC with both diagnostic and prognostic values as well as monitoring tools of treatment effectiveness.

**Figure 7:**
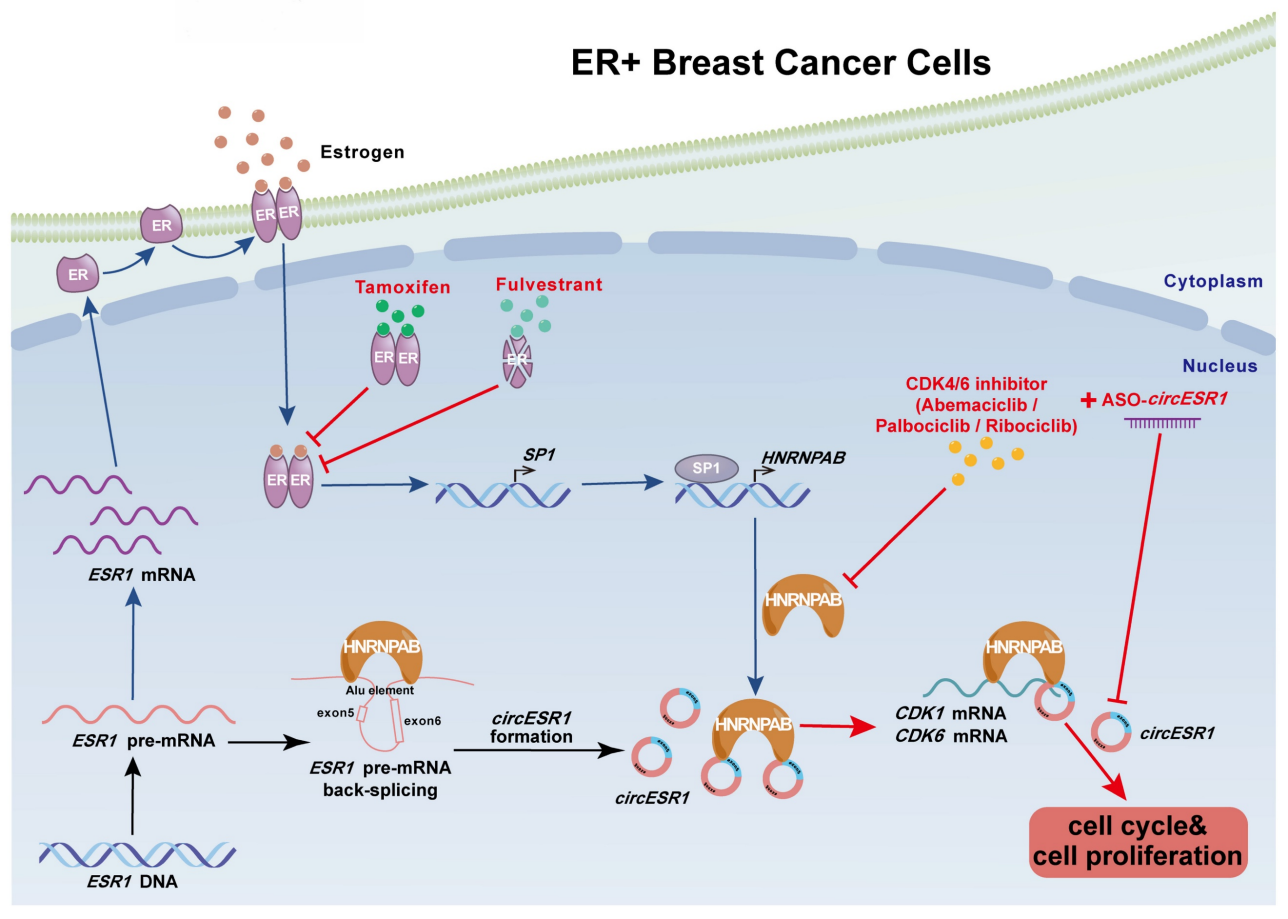
** A proposed model for the regulatory landscape of the interplay between *circESR1* and HNRNPAB and associated ER/SP1/HNRNPAB signaling axis in promoting cell cycle progression of ER+ BC.** *CircESR1* and *ESR1* mRNA were both generated from *ESR1* pre-mRNA transcript. *CircESR1* interacted with HNRNPAB, which was transcriptionally activated by estrogen secreted by fibroblasts and ER/SP1 signaling. HNRNPAB promoted the back-splicing and expression of *circESR1* by binding to the Alu elements of *ESR1* pre-mRNA; In return, *circESR1* transcripts increased the stability and expression of HNRNPAB, ensuring an efficient positive feedback loop. Further, HNRNPAB interacted and stabilized *CDK1* and *CDK6* mRNA, which was facilitated by its asymmetrical binding of *circESR1*, to promote cell cycle progression. Combined use of *circESR1* ASO and CDK4/6 inhibitors promised to be an effective therapeutic approach overcoming antiestrogen resistance in breast cancer.

In this study, *circESR1* expression was shown to be elevated in ER+ BC and further increased in tamoxifen-resistant BC cells. As *circESR1* could act both upstream and downstream of ER signaling, it might serve as an attractive therapeutic target in ER+ BC either dependent or independent of actual ERα activity. Consistently, targeting *circESR1* not only significantly increased the efficacy of tamoxifen and CDK4/6 inhibition in ER+ BC cells, but also restored the sensitivity to tamoxifen, as well as increased the efficacy of CDK4/6 inhibition in tamoxifen-resistant cells. Thus, targeting *circESR1* may afford a novel approach to improve existing therapeutic regimes for ER+ BC patients or those with de novo or acquired resistance to antiestrogen therapies. Furthermore, the synergistic efficacy exhibited by combined therapy with *circESR1* ASO and CDK4/6i against tamoxifen-resistant BC cells suggests that targeting *circESR1* might represent a promising novel approach to escalate current therapeutic regimes against ERα or CDK4/6 in ER+ patients with breast cancer (Figure 7). Notably, the preliminary data in Figure S14 revealed that CDK4/6i modulates the expression of both *circESR1* and its interacting partner HNRNPAB in ER+ BC cells. Whereas the exact mechanism requires further investigation, this finding raises the intriguing possibility that CDK4/6 inhibition may indirectly influence *circESR1*-HNRNPAB axis activity, potentially contributing to therapeutic efficacy in endocrine-resistant cancers.

## Supplementary Material

Supplementary figures and tables 1-9.

Supplementary table 10.

## Figures and Tables

**Table 1:** Association of *circESR1* RNA expression levels in tumors with the clinicopathological characteristics of ER+ BC patients.

Parameter	n	*circESR1*		*P* value
		Low expression	High expression	
ERα protein expression				< 0.0001
Low	18	17	1	
High	73	11	62	
Age (yr)				0.1712
< 60	70	19	51	
≥ 60	21	9	12	
Stage				0.0045
I	8	6	2	
II+III	83	22	61	
Lymph node metastasis				0.4660
+	42	15	27	
-	49	14	35	
Ki-67 positive ratio (%)				< 0.0001
Low (< 30)	31	18	13	
Median + High (≥ 30)	60	10	50	

P value < 0.05 in the table was marked in bold, which was regarded as statistically significant.

**Table 2:** Association of HNRNPAB protein expression levels in tumors with the clinicopathological characteristics of ER+ BC patients.

Parameter	n	HNRNPAB		*P* value
		Low expression	High expression	
ERα protein expression				0.0023
Low	18	13	5	
High	73	24	49	
Age (yr)				0.1985
< 60	70	31	39	
≥ 60	21	6	15	
Stage				0.1879
I	8	5	3	
II+III	83	32	51	
Lymph node metastasis				0.0024
+	42	10	32	
-	49	27	22	
Ki-67 positive ratio (%)				< 0.0001
Low (< 30)	31	22	9	
Median + High (≥ 30)	60	15	45	

P value < 0.05 in the table was marked in bold, which was regarded as statistically significant.

## Abbreviations

ASO: antisense oligonucleotide
CDK: cyclin-dependent kinases
circRNA: circular RNA
CLIP: crosslinking-immunprecipitation
ERα: estrogen receptor alpha
ESR1: estrogen receptor 1
hnRNP: heterogeneous nuclear ribonucleoprotein
HNRNPAB: heterogeneous nuclear ribonucleoprotein A/B
IHC: immunohistochemistry
qRT-PCR: Real-time quantitative polymerase chain reaction
RIP: RNA immunoprecipitation
RRM: RNA recognition motif
SINE: short interspersed nuclear element
TamR: tamoxifen resistant
