# Supplementary material for: The Interaction of CircESR1 and HNRNPAB Regulates Cell Cycle Transition of Breast Cancer Cell
Source: Int J Biol Sci. 2026 Jan 14;22(3):1520–41. doi: 10.7150/ijbs.126014 (PMC12839121; doi:10.7150/ijbs.126014)
Supplement: Supplementary file 1 — Supplementary figures and tables 1-9. [file ijbsv22p1520s1.pdf]

Table S1 A summary of information about the clinical characteristics, gender, age, tumor stage and molecular subtype of ER+ BC patients

Patient	Gender	Age	ER status	Stage	Lymph node metastasis	Ki-67
1	Female	33	1+	II	-	40
2	Female	59	3+	III	+	35
3	Female	42	2+	II	+	90
4	Female	58	3+	II	+	30
5	Female	28	2+	II	-	50
6	Female	43	3+	II	+	30
7	Female	50	3+	II	-	10
8	Female	44	3+	II	-	30
9	Female	50	2+	I	+	40
10	Female	45	3+	III	+	35
11	Female	50	3+	II	-	25
12	Female	30	1+	II	+	25
13	Female	37	3+	II	-	50
14	Female	65	1+	II	-	60
15	Female	50	2+	II	-	20
16	Female	36	1+	II	+	40
17	Female	41	3+	II	-	25
18	Female	42	3+	II	-	10
19	Female	47	3+	II	+	30
20	Female	67	3+	II	-	80
21	Female	65	1+	II	+	70
22	Female	50	2+	II	-	10
23	Female	66	1+	II	-	50
24	Female	50	3+	III	+	50
25	Female	49	2+	II	+	30
26	Female	52	2+	II	-	70
27	Female	46	1+	II	-	25
28	Female	38	2+	II	-	20
29	Female	46	2+	I	-	20
30	Female	79	3+	II	+	30
31	Female	58	3+	II	-	15
32	Female	66	3+	II	+	25
33	Female	57	3+	II	+	5
34	Female	48	3+	II	+	3
35	Female	49	3+	II	-	30
36	Female	47	1+	II	-	60
37	Female	52	1+	II	-	15
38	Female	53	1+	III	+	70
39	Female	48	2+	III	-	80
40	Female	59	3+	III	+	60

Table S1 (continued)

Patient	Gender	Age	ER status	Stage	Lymph node metastasis	Ki-67
41	Female	37	3+	III	+	35
42	Female	38	2+	II	+	90
43	Female	68	3+	II	+	30
44	Female	33	2+	II	-	50
45	Female	37	3+	II	+	30
46	Female	42	3+	II	-	10
47	Female	36	3+	II	-	30
48	Female	40	2+	I	+	40
49	Female	48	3+	II	-	25
50	Female	29	3+	II	-	50
51	Female	68	1+	II	+	60
52	Female	43	2+	II	-	20
53	Female	39	3+	II	-	30
54	Female	35	3+	II	-	10
55	Female	42	3+	II	+	20
56	Female	71	3+	II	-	80
57	Female	64	1+	II	+	70
58	Female	46	2+	II	-	10
59	Female	62	1+	II	-	50
60	Female	36	3+	III	+	50
61	Female	43	2+	II	+	25
62	Female	56	2+	II	-	70
63	Female	40	1+	I	-	25
64	Female	34	2+	II	-	20
65	Female	52	2+	I	-	25
66	Female	77	3+	II	+	30
67	Female	55	3+	II	-	15
68	Female	61	3+	II	+	30
69	Female	47	3+	II	+	5
70	Female	43	3+	I	+	3
71	Female	36	3+	II	-	30
72	Female	47	1+	III	-	70
73	Female	39	3+	III	+	60
74	Female	57	2+	II	-	90
75	Female	71	3+	II	+	30
76	Female	41	2+	II	-	50
77	Female	46	3+	II	-	10
78	Female	34	2+	I	+	40
79	Female	72	1+	I	+	60
80	Female	47	2+	II	-	30
81	Female	63	3+	II	-	80
82	Female	69	1+	II	+	70

Table S1 (continued)

Patient	Gender	Age	ER status	Stage	Lymph node metastasis	Ki-67
83	Female	41	2+	II	-	10
84	Female	70	1+	II	-	50
85	Female	44	3+	III	+	50
86	Female	33	2+	II	+	30
87	Female	47	2+	II	-	70
88	Female	67	3+	II	+	30
89	Female	52	3+	II	-	15
90	Female	69	3+	II	+	30
91	Female	63	3+	II	+	30

Table S2 Sequences of shRNAs

**shRNAs**	**Sense Strand (5'-3')**
sh*circESR1*-1	CCGGGGGTGCCAGGACATAACGACTGGATCCAGTCGTTATGTCCTGGCACCCTTTTG
sh*circESR1*-2	CCGGGAGGGTGCCAGGACATAACGAGGATCCTCGTTATGTCCTGGCACCCTCTTTTG
sh*circESR1*-3	CCGGGGTGCCAGGACATAACGACTAGGATCCTAGTCGTTATGTCCTGGCACCTTTTTG
sh*HNRNPAB*-1	CCGGTGGAAGCAAGTGTGAGATCAACTCGAGTTGATCTCACACTTGCTTCCATTTTTG
sh*HNRNPAB*-2	CCGGTGAGGCCATTGAATTGCCAATCTCGAGATTGGCAATTCAATGGCCTCATTTTTG
sh*HNRNPAB*-3	CCGGTGAATTGCCAATGGATCCAAACTCGAGTTTGGATCCATTGGCAATTCATTTTTG
sh*SP1*-1	CCGGGCTGGTGGTGATGGAATACATGGATCCATGTATTCCATCACCACCAGCTTTTTG
sh*SP1*-2	CCGGGCAGCAACTTGCAGCAGAATTGGATCCAATTCTGCTGCAAGTTGCTGCTTTTTG
sh*SP1*-3	CCGGCCCAAGTTTATTTCTCTCTTAGGATCCTAAGAGAGAAATAAACTTGGGTTTTTG

Table S3 Primers for cloning

**Primers for plasmid construction**	**Sense Strand (5'-3')**	**Antisense Strand (5'-3')**
circESR1 cloning primers	CGGAATTCTGAAATATGCTATCTTACAGGACATAACGACTATATGTGTCCAGC	CGGGATCCTCAAGAAAAAATATATTCACCTGGCACCCTC
circESR1-linear cloning primers	GGAATTCGACATAACGACTATATGTGTCCAGCC	CGGGATCCCTGGCACCCTCTTC
HNRNPAB cloning primers	TCCATAGAAGATTCTAGAGCCACCATGTCGGAAGCGGGCGAGGA	ATCGTCATCCTTGTAGTCGTATGGCTTGTAGTTATTCT
HNRNPAB truncation-1	CTTCTTCATAGCCATGTTCTTGCTGGCGTTG	AACGCCAGCAAGAACATGGCTATGAAGAAG
HNRNPAB truncation-2	GACTTCTTTGGGCTGCTTCATAGCCATGGC	GCCATGGCTATGAAGCAGCCCAAAGAAGTC
HNRNPAB truncation-3	GACTTCTTTGGGCTGGTTCTTGCTGGCGTTG	CAACGCCAGCAAGAACCAGCCCAAAGAAGTC
HNRNPAB promoter primers	GGGGTACCCTGAGGCAGGAGAATCGCTAGAACCTGG	CTAGCTAGCCCACGGAGCTGCTCCAACTGTGTCTC
sg*HNRNPAB* genome	CACCGTTGCAGGAGCCGCCGCGCCT	AAACAGGCGCGGCGGCTCCTGCAAC
HNRNPAB genome knock-in-1	CGCGGATCCAATAATAAAAAGGAAGAAAAAGAAAGACAAATGATTTACAGG	ATGATCTTTATAATCACCGTCATGGTCTTTGTAGTCGCTAGGACGAGGCGC
HNRNPAB genome knock-in-2	TATAAAGATCATGACATCGATTACAAGGATGACGATGACAAGATGTCGGAAGCG	CCGGAATTCAATGGTAGGGTGAGACACACGGCAACAAGTC
SP1 cloning primers	GGAATTCGCCACCATGAGCGACCAAGATC	GACCGGTGAAGCCATTGCCACTGATATTAATGGAC

Table S4 Sequences of siRNAs

**siRNAs**	**Sense Strand (5'-3')**
siNC	UUCUCCGAACGUGUCACGUTT
si*JUN*-1	CGGACCUUAUGGCUACAGUAA
si*JUN*-2	CGCAAACCUCAGCAACUUCAA
si*FOS*-1	GCGGAGACAGACCAACUAGAA
si*FOS*-2	UCUGCUUUGCAGACCGAGAUU
si*FOXA1*-1	GCGUACUACCAAGGUGUGUAU
si*FOXA1*-2	GCAGCAUAAGCUGGACUUCAA
si*SP1*-1	GCUGGUGGUGAUGGAAUACAU
si*SP1*-2	GCAGCAACUUGCAGCAGAAUU

Table S5 Sequences of sgRNA

**sgRNAs**	**Sense Strand (5'-3')**
sg*HNRNPAB*-1	CACCGCAACGCCAGCAAGAACGAGG
sg*HNRNPAB*-2	CACCGGATCAACGCCAGCAAGAACG
sg*HNRNPAB*-3	CACCGATTCCCGCTCGGGGGCGCCG

Table S6 Primers for qRT-PCR

**Primers for qRT-PCR**	**Sense Strand (5'-3')**	**Antisense Strand (5'-3')**
ACTB	CACCATTGGCAATGAGCGGTTC	AGGTCTTTGCGGATGTCCACGT
circESR1	AGGGAGCTGGTTCACATGAT	TCTCCCTCCTCTTCGGTCTT
ESR1	GCTTACTGACCAACCTGGCAGA	GGATCTCTAGCCAGGCACATTC
ACTB-converge	GTGGCCGAGGACTTTGATTG	CCTGTAACAACGCATCTCATATT
ACTB-diverge	AAAGGCGAGGCTCTGTGCT	GGGCTTACCTGTACACTGACTTGA
CDR1as-converge	TTTCCGATGGCACCTGTGTCAAG	CTGGAAGACCTTGAGATTATTGGAAGAC
CDR1as-diverge	ACGTCTCCAGTGTGCTGA	CTTGACACAGGTGCCATC
circESR1-converge	GAAGAACAGCCTGGCCTTGT	GTCTGCCAGGTTGGTCAGTA
circHIPK3	TATGTTGGTGGATCCTGTTCGGCA	TGGTGGGTAGACCAAGACTTGTGA
U1	TCCCAGGGCGAGGCTTATCCATT	GAACGCAGTCCCCCACTACCACAAAT
TFF1	CCAGTGTGCAAATAAGGGCTGC	AGGCAGATCCCTGCAGAAGTGT
GREB1	GGTCTGCCTTGCATCCTGATCT	TCCTGCTCCAAGGCTGTTCTCA
PGR	GTCGCCTTAGAAAGTGCTGTCAG	GCTTGGCTTTCATTTGGAACGCC
CDK1	GGAAACCAGGAAGCCTAGCATC	GGATGATTCAGTGCCATTTTGCC
CDK6	GGATAAAGTTCCAGAGCCTGGAG	GCGATGCACTACTCGGTGTGAA
HNRNPAB	TCCCAACACTGGACGGTCAAGA	GGGTCCTTCTTCATAGCCATGG
circESR1’s Alu-a	GTTGGATGTGGTGGGTTCCA	TGGGTGGTGACGAAGTTCAG
circESR1’s Alu-b	GACCAGCCTGACCAACAT	GGGTTCAAGCGATTCTCC
circESR1’s Alu-c	CTGTGGTCCCAGTTATGG	TATGACACGGTCTTGCTCT

Table S6 (continued)

**Primers for qRT-PCR**	**Sense Strand (5'-3')**	**Antisense Strand (5'-3')**
circESR1’s Alu-d	AGACACAGTGGCTGAAGTGA	AACGAATACACTGGGGCAGG
JUN	CCTTGAAAGCTCAGAACTCGGAG	TGCTGCGTTAGCATGAGTTGGC
FOS	GCCTCTCTTACTACCACTCACC	AGATGGCAGTGACCGTGGGAAT
FOXA1	GCAATACTCGCCTTACGGCTCT	GGGTCTGGAATACACACCTTGG
SP1	ACGCTTCACACGTTCGGATGAG	TGACAGGTGGTCACTCCTCATG

Table S7 Primary antibody used in this study

Antibody	Dilution	Catalog#	Company	Species	Purpose
ACTB	1:20000	20536-1-AP	Proteintech	Rabbit	WB
CCND1	1:500	sc-8396	Santa Cruz	Mouse	WB
CDK1	1:2000	10762-1-AP	Proteintech	Rabbit	WB
CDK1	1:500	10762-1-AP	Proteintech	Rabbit	IHC
CDK6	1:500	14052-1-AP	Proteintech	Rabbit	WB
CDK6	1:100	14052-1-AP	Proteintech	Rabbit	IHC
ERα	1:1000	#8644S	Cell Signaling Technology	Rabbit	WB
Flag	1:500	66008-4-Ig	Proteintech	Mouse	WB
Flag	2 μg	20543-1-AP	Proteintech	Rabbit	RIP, IP
HNRNPAB	1:1000	sc-376411	Santa Cruz	Mouse	WB
	1:10000	ab199724	Abcam	Rabbit	WB
HNRNPAB	2 μg	ab199724	Abcam	Rabbit	RIP
HNRNPAB	1:1000	ab199724	Abcam	Rabbit	IHC
Lamin B1	1:2000	12987-1-AP	Proteintech	Rabbit	WB
SP1	1:5000	21962-1-AP	Proteintech	Rabbit	WB
SP1	1:100	21962-1-AP	Proteintech	Rabbit	IHC
TFF1	1:500	13734-1-AP	Proteintech	Rabbit	WB
Tubulin	1:50000	66031-1-Ig	Proteintech	Mouse	WB

Table S8 Sequences of pull-down probes

Pull-down probes	Sense Strand (5'-3')
scramble	TTCTCCGAACGTGTCACGTTCGAACGTGTC
circESR1-as-biotin	TATAGTCGTTATGTCCTGGCACCCTCTTCG

Table S9 Sequences of Northern probes

Northern probes	Sense Strand (5'-3')
circESR1-as-DIG	TATAGTCGTTATGTCCTGGCACCCTCTTCG
ESR1 mRNA-DIG	GGATCTCTAGCCAGGCACATTCTAGAAGGT

Figure S1

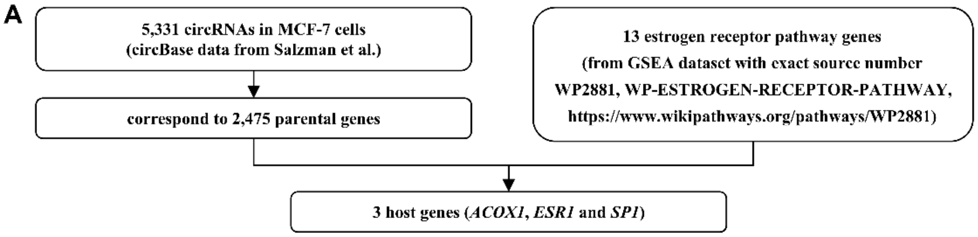

B

ACOX1	CYP1A1	CYP1A2	CYP1B1	ESR1	GPAM	JUN
NR0B2	PCK1	PDK4	PPARA	SP1	STAT3	

C

CircRNA ID	Parental gene	#Chrom	Start	End	Strand	Geneomic length	Spliced length	Samples	Best transcript	CircRNA study
hsa_circ_0045744	*ACOX1*	chr17	73944331	73949701	-	5370	1161	MCF-7	NM_001185039	Salzman2013
hsa_circ_0078309	*ESR1*	chr6	152128453	152201906	+	73453	1021	MCF-7	NM_001122740	Salzman2013
hsa_circ_0078310	*ESR1*	chr6	152201789	152265643	+	63854	453	MCF-7	NM_001122742	Salzman2013
hsa_circ_0026631	*SP1*	chr12	53803145	53803345	+	200	200	K562, A549, MCF-7	NM_138473	Salzman2013

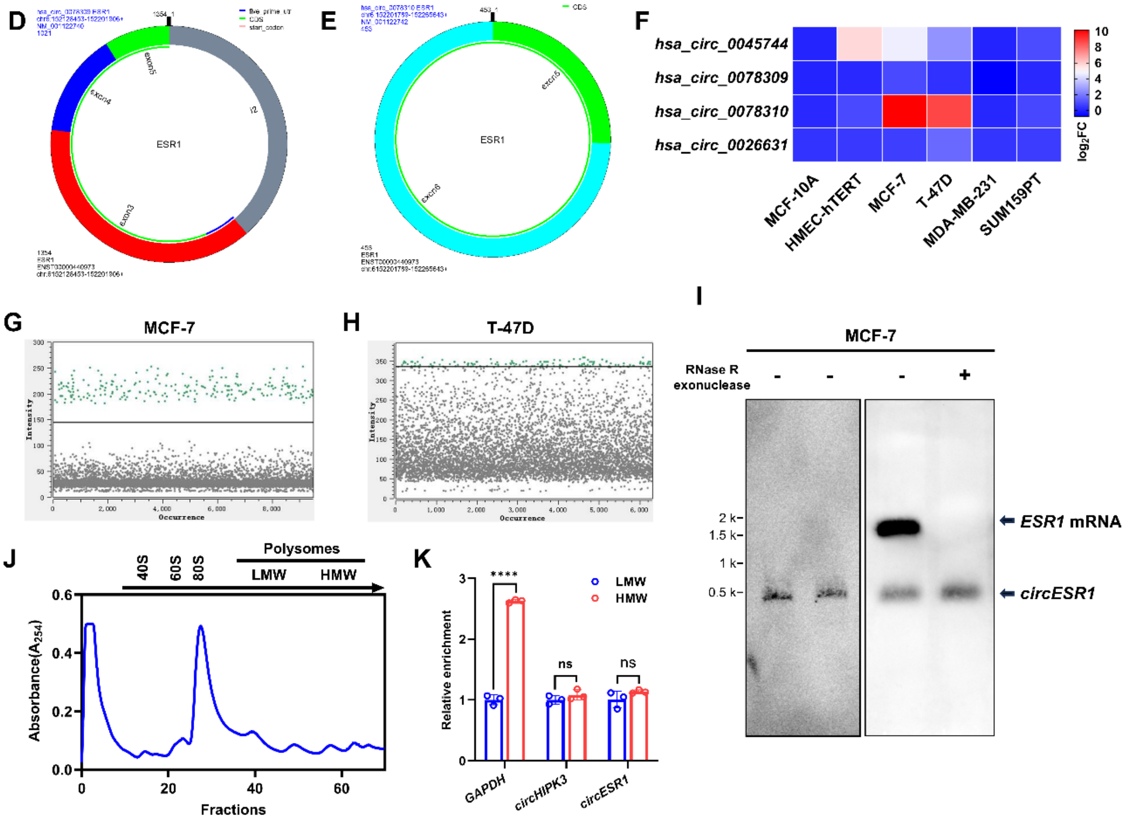

24

Figure S1 *Has_circ_0078310*, i.e. *circESR1,* is screened as a circRNA with specific expression in ER+ BC. (A) Flowchart revealing the screening of 2,475 parental genes of 5,331 circRNAs against 13 genes in the ERα pathway. (B) Matrix of 13 human genes known to function in ER signaling. Genes highlighted in red are those demonstrated to produce circRNAs. (C) Detailed annotations of four circRNAs verified to be expressed in MCF-7 cells. (D-E) The annotated parental genes in the CircBase database were two circRNAs of *ESR1*, *hsa_circ_0078309* and *hsa_circ_0078310*. The images were drawn using circPrimer 2.0 software. (F) The relative expression level of four circRNAs in six breast epithelial and carcinoma cells (MCF-10A, HMEC-hTERT, MCF-7, T-47D, MDA-MB-231 and SUM159PT) analyzed by qRT-PCR, and the log_2FC values displayed in a heatmap. (G-H) Quantification of *circESR1* copy numbers in MCF-7 and T-47D cells by digital (d)PCR. Highlighted green dots represented the detected small holes. (I) Northern blot analysis of *circESR1* in human MCF-7 cells with or without RNase R treatment. The hybridization probe against the short back-splicing junction of *circESR1* was shown (left). The hybridization probe against *ESR1* mRNA (binding to the sequences that do not overlap with *circESR1*) and against short back-splicing junction of *circESR1* was shown (right). *ESR1* mRNA served as a control for the effect of RNase R, which digests linear RNAs. (J) The curve of polysome profiling in MCF-7 cells. LMW, low molecular weight fraction; HMW, high molecular weight fraction. (K) The relative expression of *circESR1* in either LMW or HMW in MCF-7 cells analyzed by qRT-PCR. *GAPDH* mRNA was a

positive control, while *circHIPK3* was a negative control. Data was shown as mean ± S.D. from three independent experiments. Unpaired two-tailed Student's *t* test (K). ns, *P*>0.05; ****, *P*<0.0001.

Figure S2

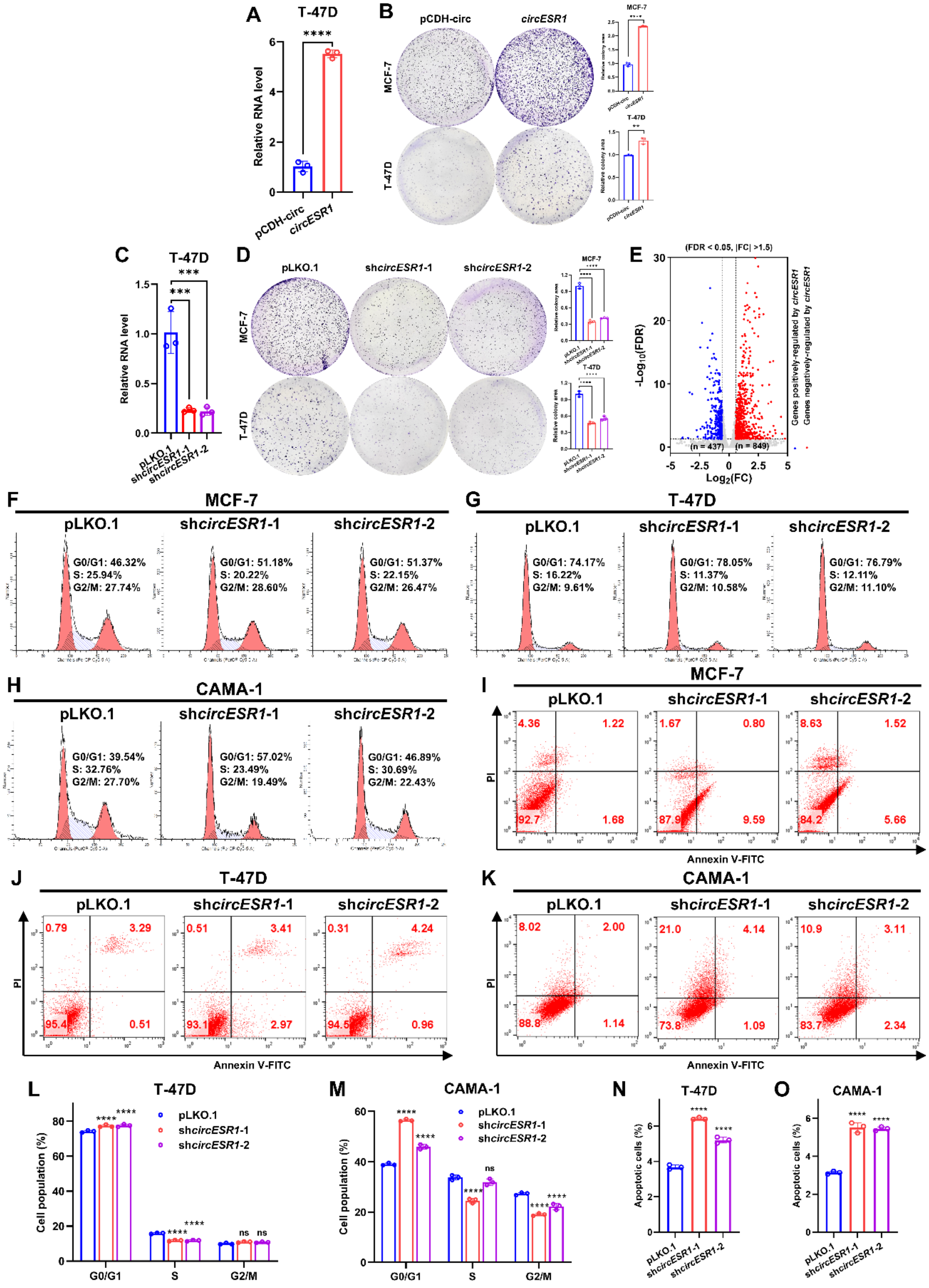
A
T-47D
Relative RNA level
pCDH-circ
circESR1
B
pCDH-circ
circESR1
MCF-7
T-47D
C
T-47D
Relative RNA level
pLKO.1
shcircESR1-1
shcircESR1-2
D
pLKO.1
shcircESR1-1
shcircESR1-2
MCF-7
T-47D
E
(FDR < 0.05, |FC| >1.5)
-Log10(FDR)
Log2(FC)
(n = 437)
(n = 849)
Genes positively-regulated by circESR1
Genes negatively-regulated by circESR1
F
MCF-7
pLKO.1
shcircESR1-1
shcircESR1-2
G0/G1: 46.32%
S: 25.94%
G2/M: 27.74%
G0/G1: 51.18%
S: 20.22%
G2/M: 28.60%
G0/G1: 51.37%
S: 22.15%
G2/M: 26.47%
G
T-47D
pLKO.1
shcircESR1-1
shcircESR1-2
G0/G1: 74.17%
S: 16.22%
G2/M: 9.61%
G0/G1: 78.05%
S: 11.37%
G2/M: 10.58%
G0/G1: 76.79%
S: 12.11%
G2/M: 11.10%
H
CAMA-1
pLKO.1
shcircESR1-1
shcircESR1-2
G0/G1: 39.54%
S: 32.76%
G2/M: 27.70%
G0/G1: 57.02%
S: 23.49%
G2/M: 19.49%
G0/G1: 46.89%
S: 30.69%
G2/M: 22.43%
I
MCF-7
pLKO.1
shcircESR1-1
shcircESR1-2
PI
Annexin V-FITC
4.36
1.22
92.7
1.68
1.67
0.80
87.9
9.59
8.63
1.52
84.2
5.66
J
T-47D
pLKO.1
shcircESR1-1
shcircESR1-2
PI
Annexin V-FITC
0.79
3.29
95.4
0.51
0.51
3.41
93.1
2.97
0.31
4.24
94.5
0.96
K
CAMA-1
pLKO.1
shcircESR1-1
shcircESR1-2
PI
Annexin V-FITC
8.02
2.00
88.8
1.14
21.0
4.14
73.8
1.09
10.9
3.11
83.7
2.34
L
T-47D
Cell population (%)
pLKO.1
shcircESR1-1
shcircESR1-2
G0/G1
S
G2/M
M
CAMA-1
Cell population (%)
pLKO.1
shcircESR1-1
shcircESR1-2
G0/G1
S
G2/M
N
T-47D
Apoptotic cells (%)
pLKO.1
shcircESR1-1
shcircESR1-2
O
CAMA-1
Apoptotic cells (%)
pLKO.1
shcircESR1-1
shcircESR1-2

48

Figure S2 *CircESR1* promotes cell cycle transition and reduces cell apoptosis. (A) The relative expression of *circESR1* in T-47D cells bearing control vector or vector expressing *circESR1* analyzed by qRT-PCR. (B) Cell viability in MCF-7 and T-47D cells bearing control vector or vector expressing *circESR1* determined by colony formation assay. (C) The relative expression of *circESR1* in T-47D cells bearing control or *circESR1* shRNAs analyzed by qRT-PCR. (D) Cell viability in MCF-7, and T-47D cells bearing control or *circESR1* shRNAs determined by colony formation assays. (E) Genes that are positively and negatively regulated by *circESR1* are shown by volcano plot (FDR < 0.05, |FC| > 1.5). (F-H and L-M) Flow cytometry showed the cell cycle distribution of ER+ BC cells bearing control or *circESR1* shRNAs. (I-K and N-O) Flow cytometry showed total proportion of cell apoptosis (Annexin V-FITC+) in ER+BC cells bearing control or *circESR1* shRNAs. Data was shown as mean ± S.D. from three independent experiments. Unpaired two-tailed Student's *t* test (A-B) and one-way ANOVA followed by Tukey's multiple comparisons test (C-D, L-O). ns, $P>0.05$; **, $P<0.01$; ***, $P<0.001$; ****, $P<0.0001$.

Figure S3

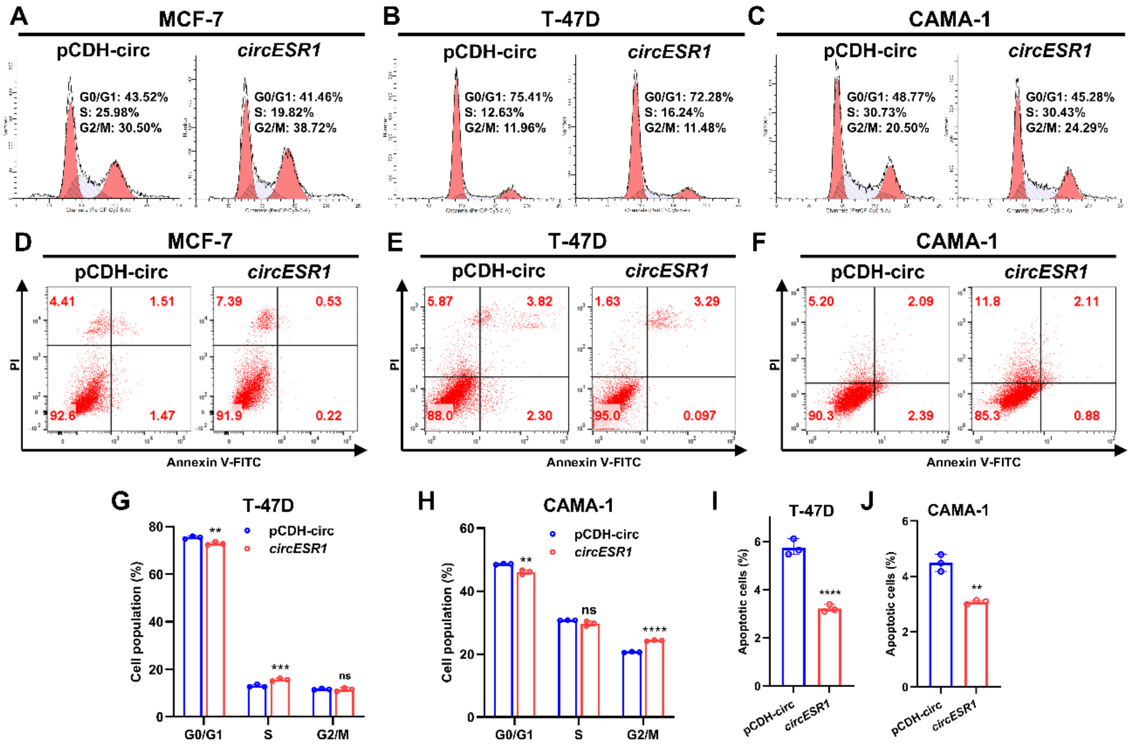

Figure S3 *CircESR1* promotes cell cycle transition and reduces cell apoptosis. (A-C and G-H) Flow cytometry showed the cell cycle distribution of ER+ BC cells bearing control vector or vector expressing *circESR1*. (D-F and I-J) Flow cytometry showed total proportion of cell apoptosis (Annexin V-FITC+) in ER+ BC cells bearing control vector or vector expressing *circESR1*. Data was shown as mean ± S.D. from three independent experiments. Unpaired two-tailed Student's *t* test (G-J). ns, *P*>0.05; **, *P*<0.01; ***, *P*<0.001; ****, *P*<0.0001.

Figure S4

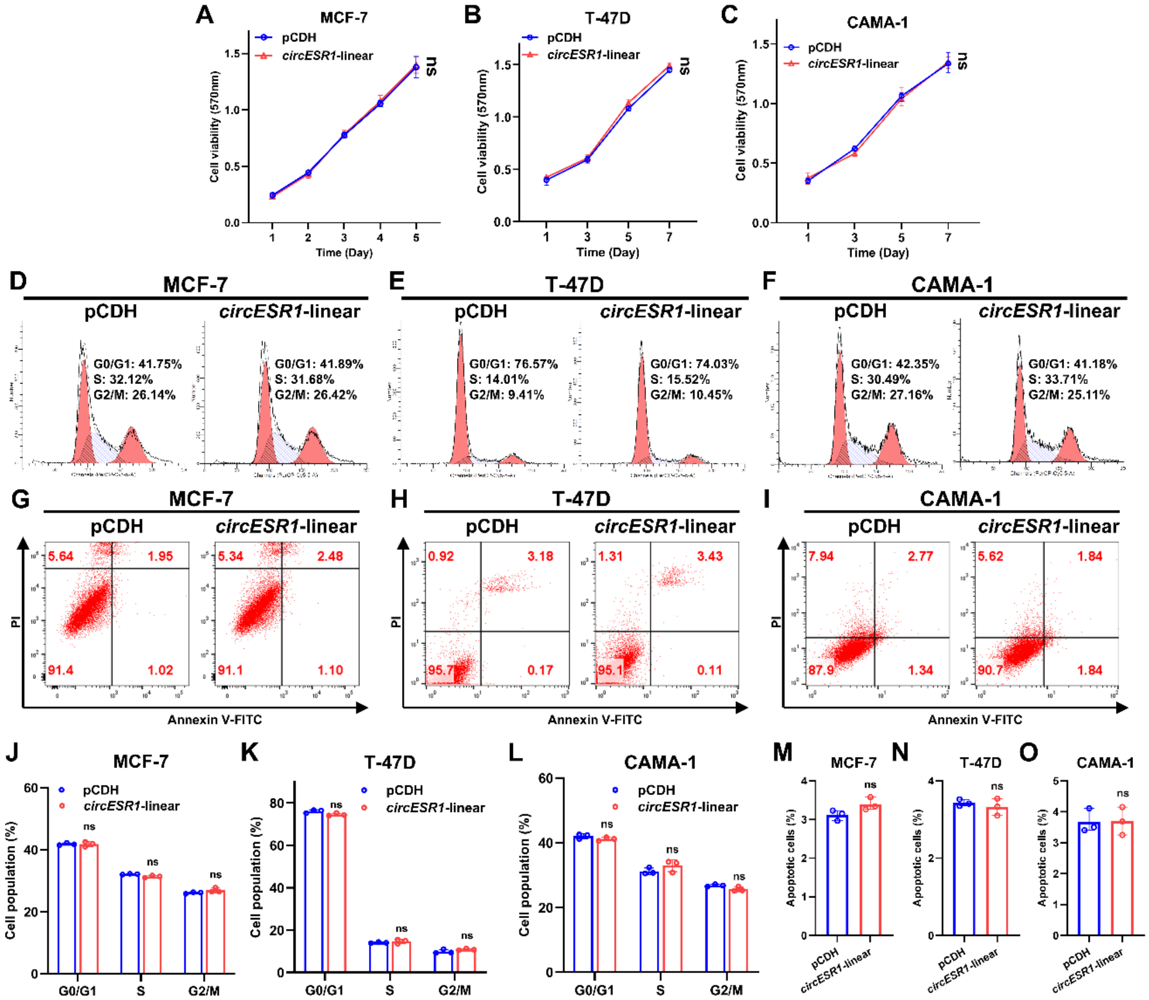

Figure S4 *CircESR1* cognate linear sequences have no effect on cell cycle transition and cell apoptosis. (A-C) Cell viability in ER+ BC cells bearing control vector or vector expressing *circESR1* cognate linear sequences determined by MTT assay. (D-F and J-L) Flow cytometry showed the cell cycle distribution of ER+ BC cells bearing control vector or vector expressing *circESR1* cognate linear sequences. (G-I and M-O) Flow cytometry showed total proportion of cell apoptosis (Annexin V-FITC+) in ER+ BC cells bearing control vector or vector expressing *circESR1* cognate linear sequences. Data was shown

as mean ± S.D. from three independent experiments. Unpaired two-tailed Student's *t* test (J-O) and two-way ANOVA test (a-c). ns, $P>0.05$.

Figure S5

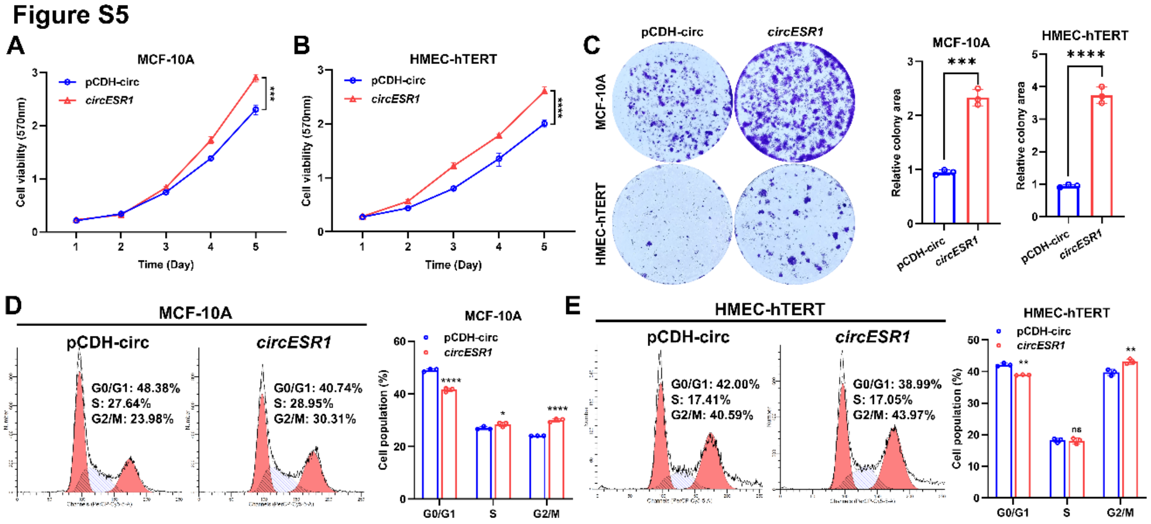

Figure S5 *CircESR1* promotes cell cycle transition in normal epithelial breast cells. (A-B) Cell viability in ER- mammary epithelial cells MCF-10A and HMEC-hTERT bearing control vector or vector expressing *circESR1* determined by MTT assay. (C) Cell viability in MCF-10A and HMEC-hTERT cells bearing control vector or vector expressing *circESR1* determined by colony formation assay. (D-E) Flow cytometry showed the cell cycle distribution and total proportion of cell apoptosis (Annexin V-FITC+) in MCF-10A and HMEC-hTERT cells bearing control vector or vector expressing *circESR1*. Data was shown as mean ± S.D. from three independent experiments. Unpaired two-tailed Student's *t* test (C-E) and two-way ANOVA test (A-B). ns, P>0.05; *, P<0.05; **, P<0.01; ***, P<0.001; ****, P<0.0001.

Figure S6

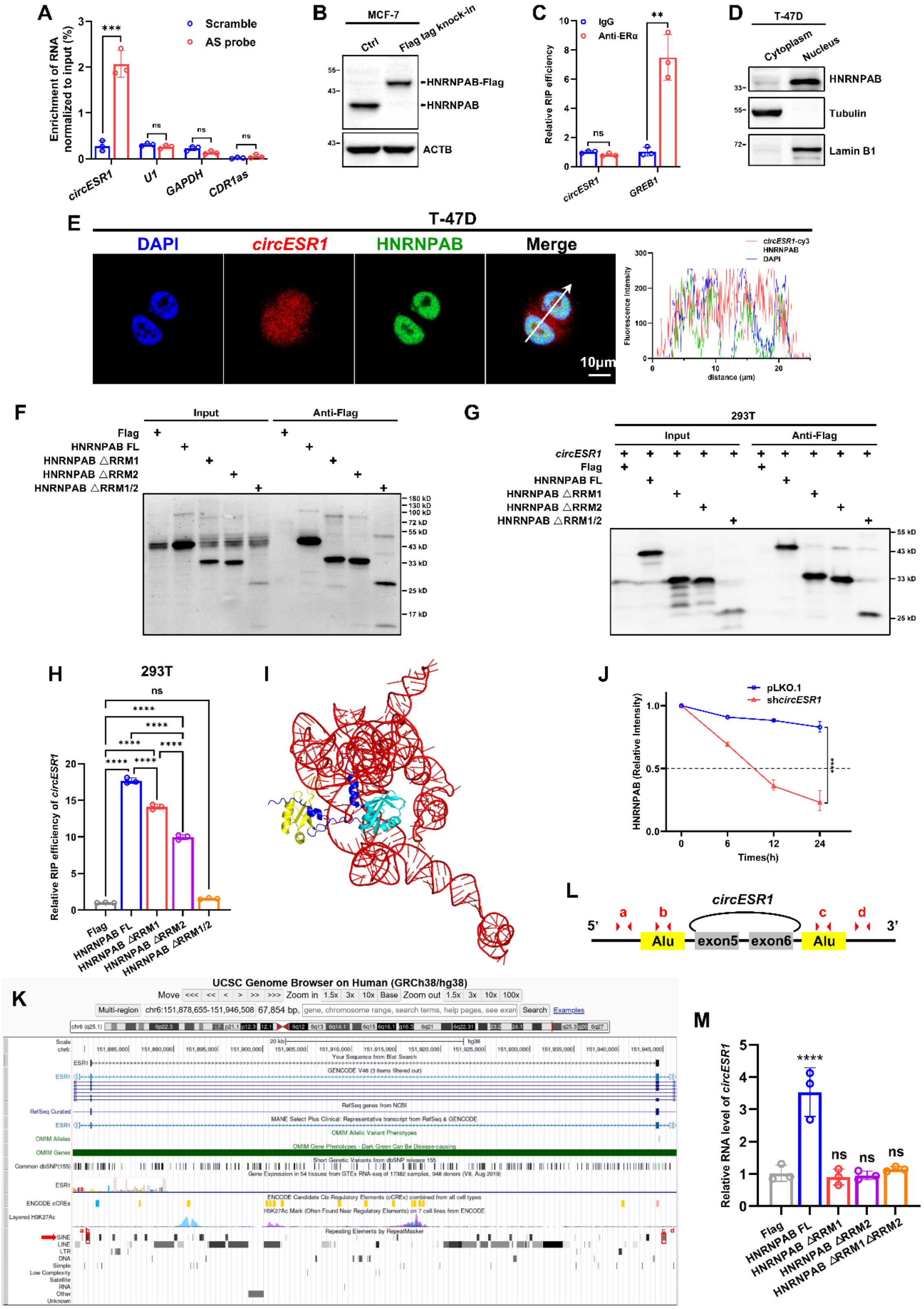
A
Enrichment of RNA normalized to input (%)
Scramble
AS probe
***
ns
circESR1
U1
GAPDH
CDR1as
B
MCF-7
Ctrl
Flag tag knock-in
HNRNPAB-Flag
HNRNPAB
ACTB
C
IgG
Anti-ERα
**
ns
Relative RIP efficiency
circESR1
GREB1
D
T-47D
Cytoplasm
Nucleus
HNRNPAB
Tubulin
Lamin B1
E
T-47D
DAPI
circESR1
HNRNPAB
Merge
10μm
circESR1-cy3
HNRNPAB
DAPI
Fluorescence Intensity
distance (μm)
F
Input
Anti-Flag
Flag
HNRNPAB FL
HNRNPAB △RRM1
HNRNPAB △RRM2
HNRNPAB △RRM1/2
G
293T
Input
Anti-Flag
circESR1
Flag
HNRNPAB FL
HNRNPAB △RRM1
HNRNPAB △RRM2
HNRNPAB △RRM1/2
H
293T
ns
****
Relative RIP efficiency of circESR1
Flag
HNRNPAB FL
HNRNPAB ΔRRM1
HNRNPAB ΔRRM2
HNRNPAB ΔRRM1/2
I
J
pLKO.1
shcircESR1
HNRNPAB (Relative Intensity)
****
Times(h)
K
UCSC Genome Browser on Human (GRCh38/hg38)
L
circESR1
5'
3'
a
b
c
d
Alu
exon5
exon6
Alu
M
Relative RNA level of circESR1
****
ns
Flag
HNRNPAB FL
HNRNPAB ΔRRM1
HNRNPAB ΔRRM2
HNRNPAB ΔRRM1ΔRRM2

93

Figure S6 *CircESR1* interacts with HNRNPAB and HNRNPAB promoted the biogenesis of *circESR1*. (A) Efficiency of enriching *circESR1* with AS probe was evaluated in RNA pull-down experiment. *U1*, *GAPDH* mRNA and *CDR1as* as negative control. (B) Immunoblot assessment of HNRNPAB expression in MCF-7 cells or cells with CRISPR knock-in 3xFlag tag in the N-terminal of HNRNPAB. (C) The relative expression of *circESR1* and *GREB1* mRNA using IgG and anti-ERα antibodies to perform RIP assay in MCF-7 cells. (D) Detected the cellular subcellular localization of HNRNPAB after nuclear and cytoplasmic separation of T-47D cells. (E) Immunofluorescence analysis localization of DAPI (blue), *circESR1 AS* probe labeled with cy3 (red) and anti-HNRNPAB antibody (green) in T-47D cells. Scale bars, 10 μm. (F) Immunoblot detected RIP results after transiently transferring different truncated mutation vectors of HNRNPAB using anti-Flag antibodies in MCF-7 cells. (G-H) Immunoblot and qRT-PCR detected RIP results after transiently transfecting different truncated mutation vectors of HNRNPAB using anti-Flag antibodies into 293T cells. (I) The predicted 3D model of *circESR1* (red) and HNRNPAB (blue) by HDOCK software. Highlighted yellow or cyan represented the two RRM domains of HNRNPAB. (J) The relative intensity analysis of the immunoblot results in Fig. 3o using Image J software. (K) The UCSC Genome Browser searched for Alu sequences flanking *circESR1* based on genomic location. Highlighted red represented the two SINE (short interspersed element) sequences. (L) The schematic of Alu sequence on both sides of the upstream and downstream of

circESR1 in *ESR1* pre-mRNA (b and c regions). The regions outside of Alu sequence on both sides (a and d regions) were chosen as negative controls. (M) The relative expression of *circESR1* in MCF-7 cells bearing Flag vector or different truncated mutation vectors of HNRNPAB analyzed by qRT-PCR. Data was shown as mean ± S.D. from three independent experiments. Unpaired two-tailed Student's *t* test (A, C) and one-way ANOVA followed by Tukey's multiple comparisons test (H, M) and two-way ANOVA test (J). ns, $P>0.05$; **, $P<0.01$; ***, $P<0.001$; ****, $P<0.0001$.

Figure S7

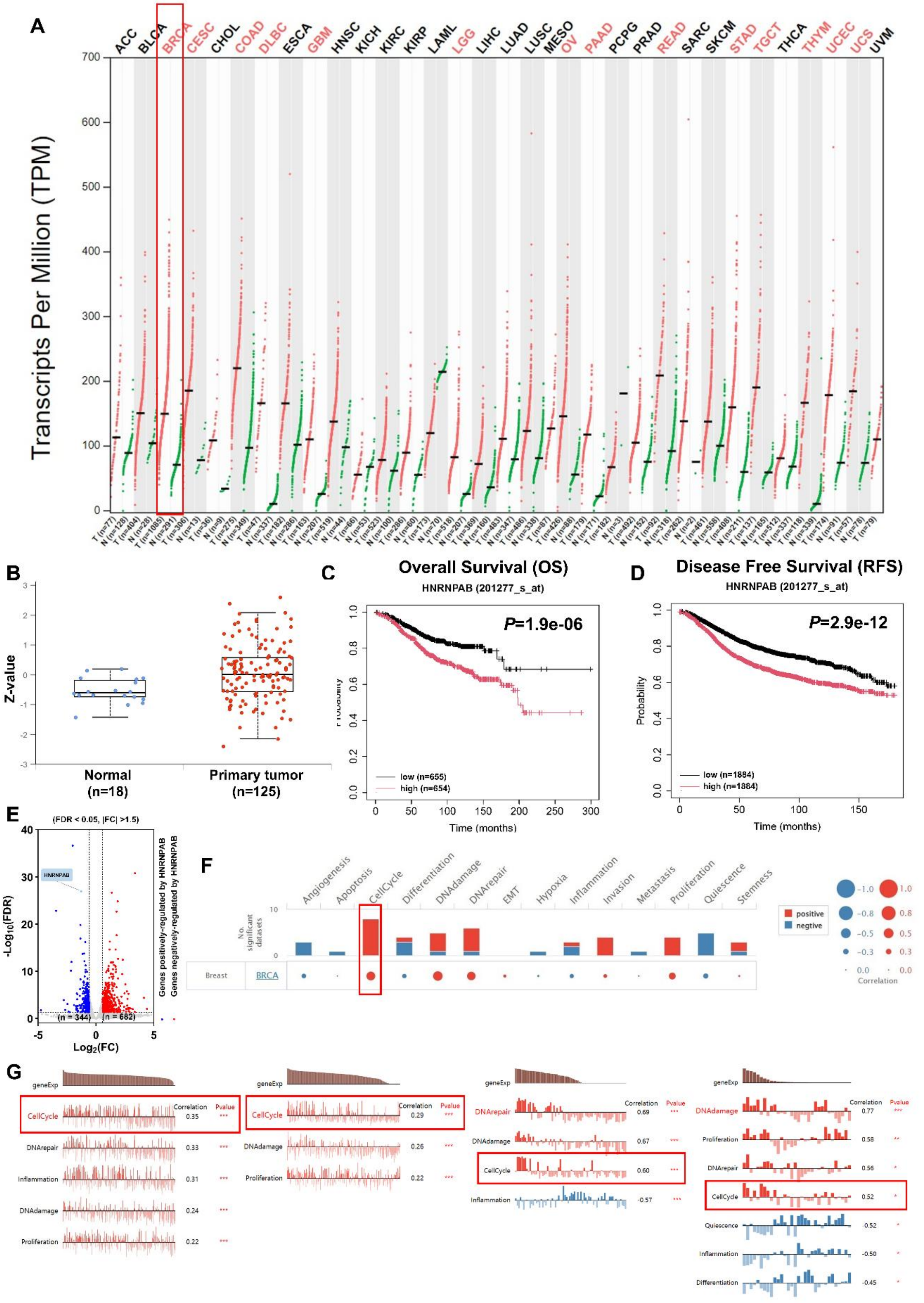

121

Figure S7 Analysis of HNRNPAB expression by bioinformatics. (A) GEPIA2 database analysis of *HNRNPAB* mRNA expression levels in 33 different types of cancer compared to adjacent tissues. The cancer names are marked in red, indicating significant differences in *HNRNPAB* mRNA among this cancer type. (B) Expression level of *HNRNPAB* in normal breast tissue (n=18) and primary BC tissue (n=125). (C-D) KM Plotter analyzed the difference in OS and PFS of ER+ BC patients with different levels of *HNRNPAB* expression. *P* value was determined by Pearson correlation analysis. (E) Genes that are positively and negatively regulated by HNRNPAB are shown by volcano plot (FDR < 0.05, |FC| > 1.5). (F) The CancerSEA database had analyzed the different functional correlations of *HNRNPAB* in 18 different cancer types at the single cell level, and the image analysis showed the correlation with the functional status of BC. (G) GSE77308, GSE75688, GSE75367, GSE86978 dataset analyzed the functionality of HNRNPAB and only displayed functions with correlation absolute values more than 0.2 and significant *P*-values. *, *P*<0.05; **, *P*<0.01; ***, *P*<0.001.

Figure S8

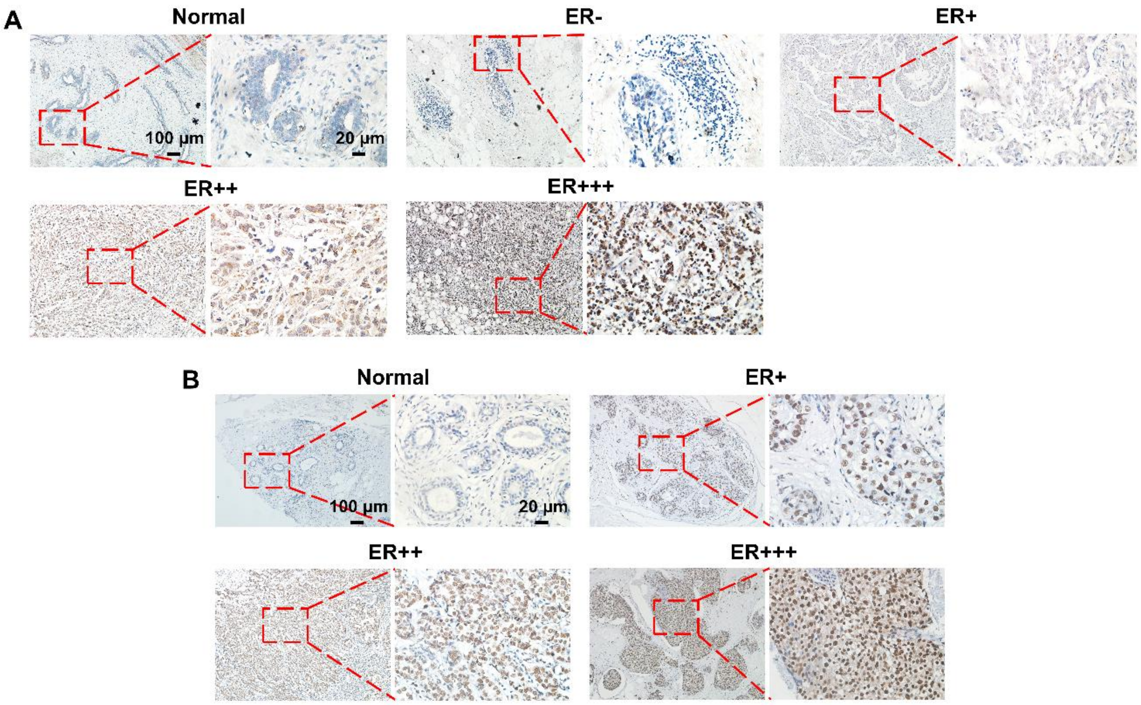

Figure S8 Examination of *circESR1* and HNRNPAB expression in clinical samples. (A) *In situ* hybridization staining of *circESR1* was performed on the tissue sections of clinical BC patients with normal and different levels of ER protein expression, and the staining of *circESR1* was observed by Olympus inverted fluorescence microscope IX73. The left scale bar of each group is 100 μm, and the right scale bar is 20 μm. (B) IHC staining of HNRNPAB protein was performed on the tissue sections of clinical BC patients with normal and different levels of ER protein expression, and the expression of HNRNPAB protein was observed by Olympus inverted fluorescence microscope IX73. The left scale bar of each group is 100 μm, and the right scale bar is 20 μm.

Figure S9

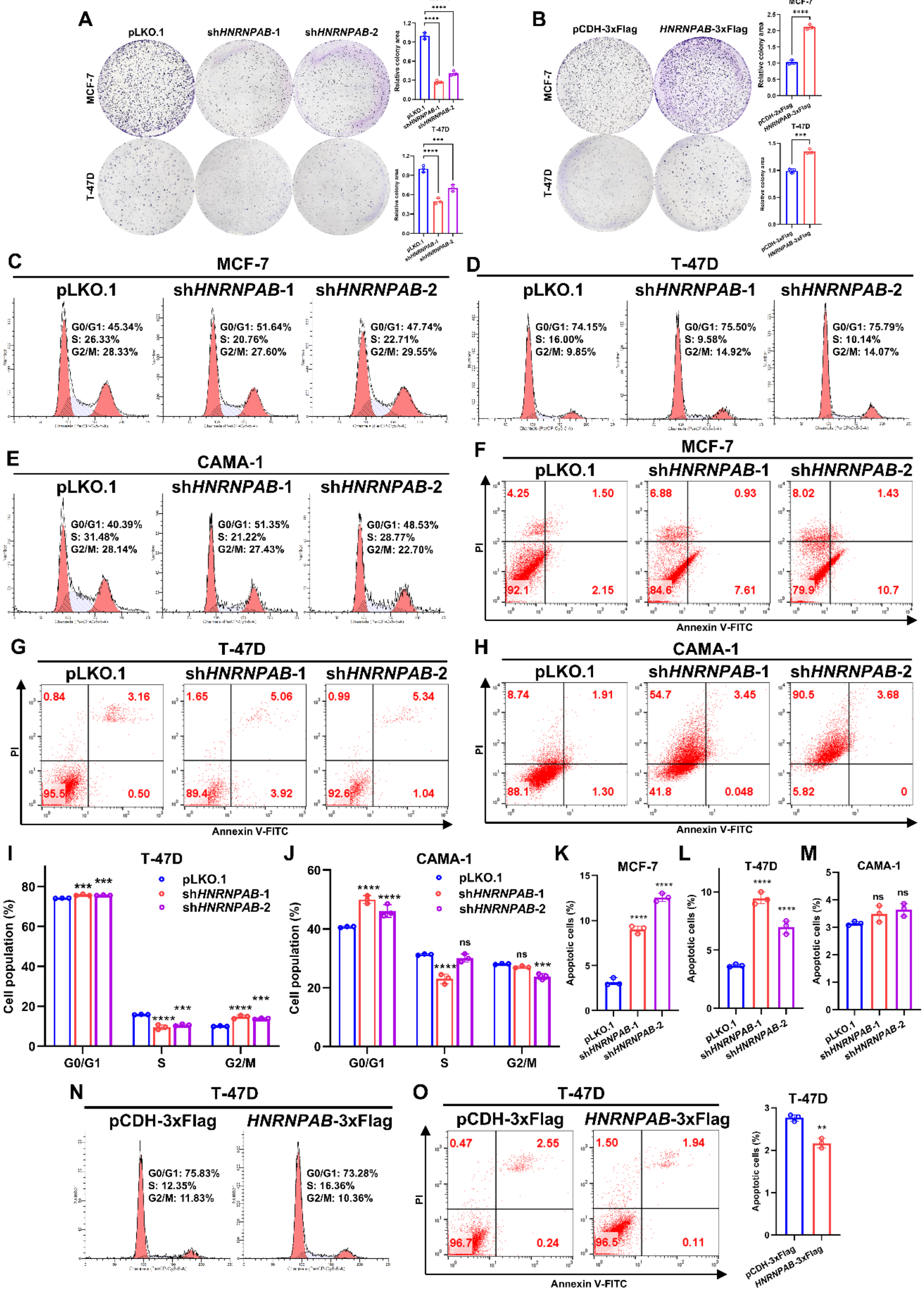
A
pLKO.1
shHNRNPAB-1
shHNRNPAB-2
MCF-7
T-47D
Relative colony area
****
***
B
pCDH-3xFlag
HNRNPAB-3xFlag
MCF-7
T-47D
Relative colony area
C
MCF-7
pLKO.1
G0/G1: 45.34%
S: 26.33%
G2/M: 28.33%
shHNRNPAB-1
G0/G1: 51.64%
S: 20.76%
G2/M: 27.60%
shHNRNPAB-2
G0/G1: 47.74%
S: 22.71%
G2/M: 29.55%
D
T-47D
pLKO.1
G0/G1: 74.15%
S: 16.00%
G2/M: 9.85%
shHNRNPAB-1
G0/G1: 75.50%
S: 9.58%
G2/M: 14.92%
shHNRNPAB-2
G0/G1: 75.79%
S: 10.14%
G2/M: 14.07%
E
CAMA-1
pLKO.1
G0/G1: 40.39%
S: 31.48%
G2/M: 28.14%
shHNRNPAB-1
G0/G1: 51.35%
S: 21.22%
G2/M: 27.43%
shHNRNPAB-2
G0/G1: 48.53%
S: 28.77%
G2/M: 22.70%
F
MCF-7
pLKO.1
4.25
1.50
92.1
2.15
shHNRNPAB-1
6.88
0.93
84.6
7.61
shHNRNPAB-2
8.02
1.43
79.9
10.7
PI
Annexin V-FITC
G
T-47D
pLKO.1
0.84
3.16
95.5
0.50
shHNRNPAB-1
1.65
5.06
89.4
3.92
shHNRNPAB-2
0.99
5.34
92.6
1.04
PI
Annexin V-FITC
H
CAMA-1
pLKO.1
8.74
1.91
88.1
1.30
shHNRNPAB-1
54.7
3.45
41.8
0.048
shHNRNPAB-2
90.5
3.68
5.82
0
PI
Annexin V-FITC
I
T-47D
pLKO.1
shHNRNPAB-1
shHNRNPAB-2
Cell population (%)
G0/G1
S
G2/M
J
CAMA-1
pLKO.1
shHNRNPAB-1
shHNRNPAB-2
Cell population (%)
G0/G1
S
G2/M
ns
K
MCF-7
Apoptotic cells (%)
L
T-47D
Apoptotic cells (%)
M
CAMA-1
Apoptotic cells (%)
ns
N
T-47D
pCDH-3xFlag
G0/G1: 75.83%
S: 12.35%
G2/M: 11.83%
HNRNPAB-3xFlag
G0/G1: 73.28%
S: 16.36%
G2/M: 10.36%
O
T-47D
pCDH-3xFlag
0.47
2.55
96.7
0.24
HNRNPAB-3xFlag
1.50
1.94
96.5
0.11
PI
Annexin V-FITC
T-47D
Apoptotic cells (%)
**

Figure S9 Analysis of cell proliferation, cell cycle and cell apoptosis upon the manipulation of HNRNPAB expression. (A-B) Cell viability in MCF-7 and T-47D cells bearing control or *HNRNPAB* shRNAs (A) or vector expressing *HNRNPAB* (B) determined by colony formation assay. (C-E and I-J) Flow cytometry showed the cell cycle distribution of ER+ BC cells bearing control or *HNRNPAB* shRNAs. (F-H and K-M) Flow cytometry showed total proportion of cell apoptosis (Annexin V-FITC+) in ER+ BC cells bearing control or *HNRNPAB* shRNAs. (N-O) Flow cytometry showed the cell cycle distribution and total proportion of cell apoptosis (Annexin V-FITC+) in T-47D cells bearing control vector or vector expressing HNRNPAB. Data was shown as mean ± S.D. from three independent experiments. Unpaired two-tailed Student's t test (B, O) and one-way ANOVA followed by Tukey's multiple comparisons test (A, I-M). ns, $P>0.05$; **, $P<0.01$; ***, $P<0.001$; ****, $P<0.0001$.

Figure S10

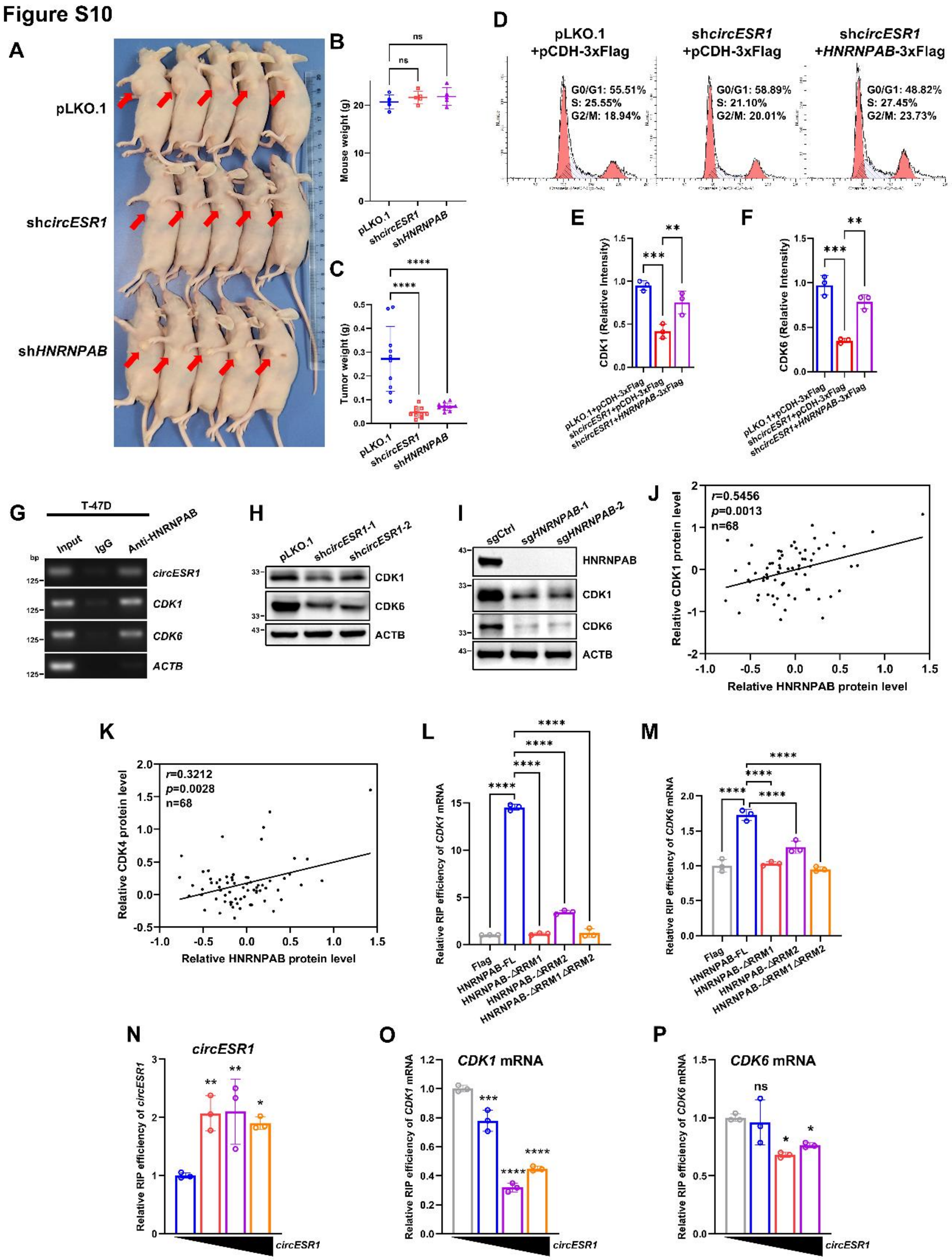

159

160 Figure S10 HNRNPAB promotes cell cycle transition by interacting with *CDK1* and

161 *CDK6* mRNA. (A-C) Female BALB/c nude mice injected with 2×10^6 MCF-7 control or

knockdown *circESR1* or knockdown *HNRNPAB* cells (n=5/group). On the 48th day, when collecting tumors from mice, weigh the live mice and the final weight of the isolated tumors. (D) Flow cytometry showed the cell cycle distribution of MCF-7 cells bearing control or *circESR1* shRNAs in the presence or absence of vector expressing HNRNPAB. (E-F) The relative intensity analysis of the immunoblot results in Figure 4I using Image J software. (G) The mRNA enriched by HNRNPAB in RIP-seq was verified in T-47D cells, and was displayed by agarose gel electrophoresis after analyzed by qRT-PCR. (H-I) Immunoblot assessed the expression of CDK1 and CDK6 proteins after *circESR1* depletion or *HNRNPAB* knockout in MCF-7 cells. (J-K) CPTAC database analyzed relative HNRNPAB and CDK1 or CDK4 protein levels in 68 ER+ BC patients. (L-M) 293T cells were transiently transfected with HNRNPAB plasmids with different truncations and *circESR1* plasmid. RIP assay by use of anti-Flag antibody and the relative expression of *CDK1* and *CDK6* mRNA analyzed by qRT-PCR. (N-P) 293T cells co-transfected with the RRM2 domain deletion of HNRNPAB (15 μg) and gradient amounts of *circESR1* (1, 5, 9, 13 μg) were used for RIP assay by use of anti-Flag antibody, then the relative expression of *circESR1* and *CDK1, CDK6* mRNAs analyzed by qRT-PCR. Data was shown as mean ± S.D. from three independent experiments. One-way ANOVA followed by Tukey's multiple comparisons test (B-C, E-F, L-P). ns, $P>0.05$; *, $P<0.05$; **, $P<0.01$; ***, $P<0.001$; ****, $P<0.0001$.

Figure S11

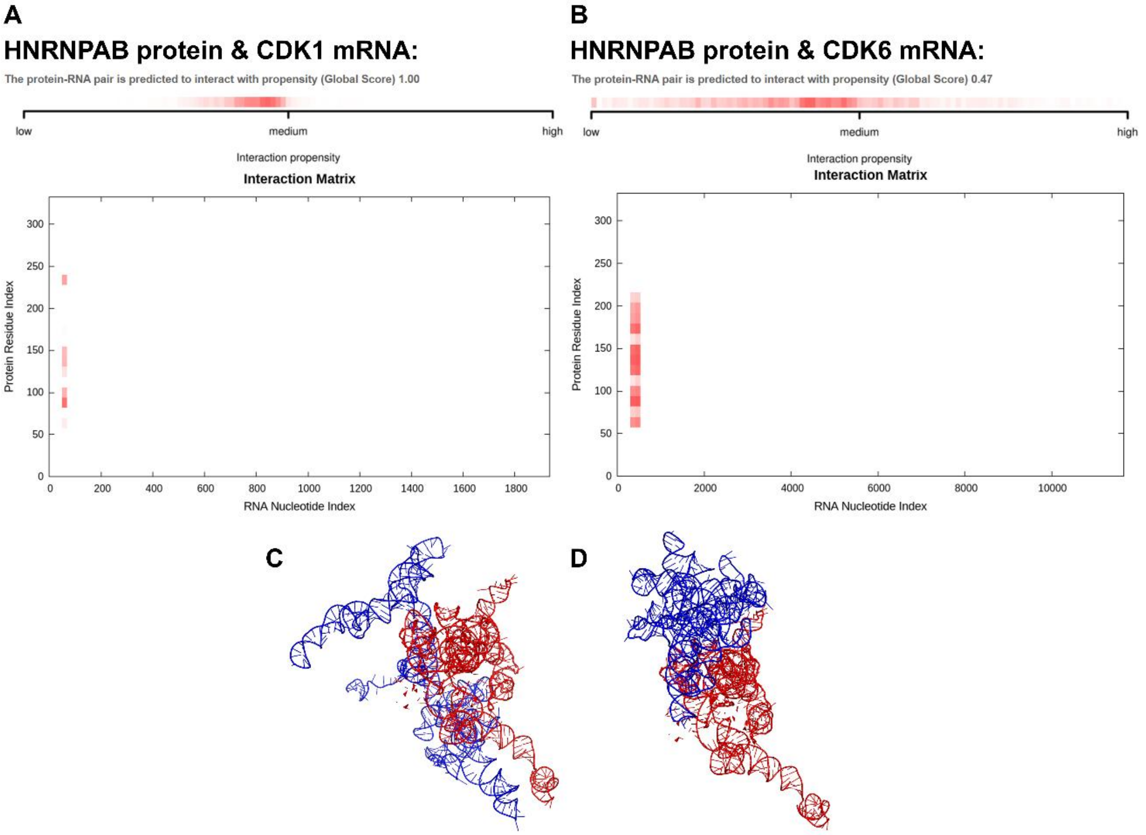

E

Score	Expect	Method	Identities	Positives	Gaps
60.5 bits(145)	2e-19	Compositional matrix adjust.	28/68(41%)	47/68(69%)	1/68(1%)

```
Query  1   KMFVGGLSWDT-SKKDLKDYFTKFGEVVDCTIKMDPNTGRSRGFGFILFKDAASVEKVLD  59
           K+FVGGL+ ++ +++ +++YF +FGE+     + MDP    + RGF FI FK+    V+KVL+
Sbjct  1   KIFVGGLNPESPTEEKIREYFGEFGEIEAIELPMDPKLNKRRGFVFITFKEEEPVKKVLE  60

Query  60  QKEHRLDG  67
           +K H + G
Sbjct  61  KKFHTVSG  68
```

Figure S11 Molecular docking analysis of HNRNPAB and *CDK1* or *CDK6* mRNAs. (A) The interaction propensities between the amino acid sequence of HNRNPAB and *CDK1* mRNA were predicted using the catRAPID fragments tool. The horizontal axis in the figure below corresponds to the *CDK1* mRNA sequence, and the vertical axis corresponds to the HNRNPAB amino acid sequence. (B) The predicted interaction propensities between the amino acid sequence of HNRNPAB and *CDK6* mRNA using the catRAPID fragments tool. The horizontal axis in the figure below corresponds to the

CDK6 mRNA sequence, and the vertical axis corresponds to the HNRNPAB amino acid sequence. (C-D) The predicted 3D model of *circESR1* and HNRNPAB complex (red) with *CDK1* or *CDK6* mRNAs (blue) by HDOCK software. Highlighted red represented the whole structure of previous *circESR1* and HNRNPAB complex (Figure S6I). (E) Alignment of the two predicted RRM domain of HNRNPAB using NCBI blast tool.

Figure S12

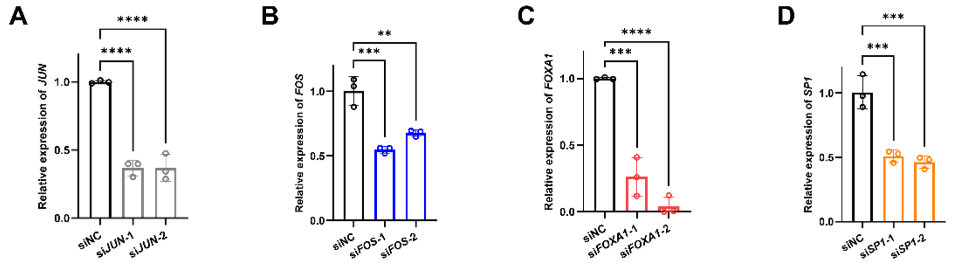

Figure S12 Estrogen promotes HNRNPAB expression via SP1. (A-D) MCF-7 cells were transfected with pGL3 basic reporter vector containing HNRNPAB promoter, pRL-TK reporter control vector containing luciferase activity, as well as siRNA control or different siRNA sequences of 4 transcription factors (JUN, FOS, FOXA1 and SP1), then the relative expression of each transcription factors analyzed by qRT-PCR. Data was shown as mean ± S.D. from three independent experiments. One-way ANOVA followed by Tukey's multiple comparisons test (A-D). **, $P<0.01$; ***, $P<0.001$; ****, $P<0.0001$.

Figure S13

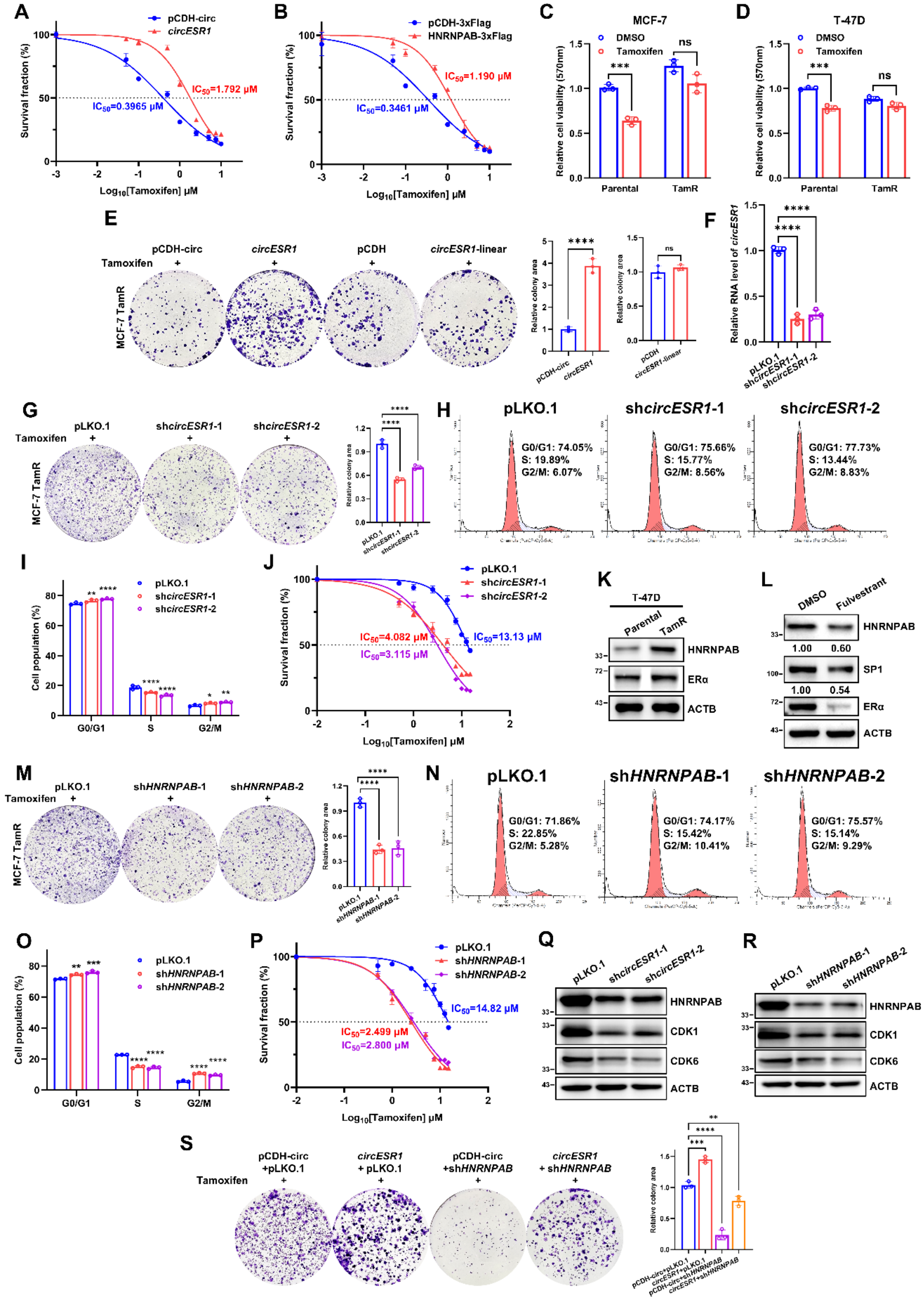

202

Figure S13 *CircESR1* promotes antiestrogen resistance via HNRNPAB. (A-B) MCF-7 cells bearing control vector or vector expressing *circESR1* or *HNRNPAB* were treated with different concentration gradients of tamoxifen for 7 days. The drug killing curve of the cells was detected by MTT assay. (C-D) Cell viability in MCF-7 and T-47D parental and tamoxifen resistant (TamR) cells treated with 1 μM tamoxifen or DMSO vehicle for 7 days determined by MTT assay. (E) Cell viability in MCF-7 TamR cells bearing control vector or vector expressing *circESR1* or cognate linear sequences determined by colony formation assay. (F) The relative expression of *circESR1* and *ESR1* mRNA in MCF-7 TamR cells bearing control or *circESR1* shRNAs analyzed by qRT-PCR. (G) Cell viability in MCF-7 TamR cells bearing control or *circESR1* shRNAs determined by colony formation assays. (H-I) Flow cytometry showed the cell cycle distribution of MCF-7 TamR cells bearing control or *circESR1* shRNAs. (J) MCF-7 cells bearing control or *circESR1* shRNAs were treated with different concentration gradients of tamoxifen for 7 days. The drug killing curve of the cells detected by MTT assay. (K) Immunoblot assessed the expression of HNRNPAB and ERα proteins in T-47D parental and TamR cells. (L) Immunoblot assessed the expression of HNRNPAB, SP1 and ERα proteins in MCF-7 TamR cells treated with DMSO or 10 mM fulvestrant for 48 h. (M) Cell viability in MCF-7 TamR cells bearing control or *HNRNPAB* shRNAs determined by colony formation assay. (N-O) Flow cytometry showed the cell cycle distribution of MCF-7 TamR cells bearing control or *HNRNPAB* shRNAs. (P) MCF-7 cells bearing control or

HNRNPAB shRNAs treated with different concentration gradients of tamoxifen for 7 days. The drug killing curve of the cells detected by MTT assay. (Q-R) Immunoblot assessed the expression of HNRNPAB, CDK1 and CDK6 proteins after *circESR1* or *HNRNPAB* depletion in MCF-7 TamR cells. (S) Cell viability in MCF-7 TamR cells bearing control vector or vector expressing *circESR1* in the absence or presence of HNRNPAB shRNA with 1 μM tamoxifen treatment determined by colony formation assay. Data was shown as mean ± S.D. from three independent experiments. Unpaired two-tailed Student's *t* test (C-E) and one-way ANOVA followed by Tukey's multiple comparisons test (F-G, I, M, O, S) and two-way ANOVA test (A-B, J, P). ns, $P>0.05$; *, $P<0.05$; **, $P<0.01$; ***, $P<0.001$; ****, $P<0.0001$.

Figure S14

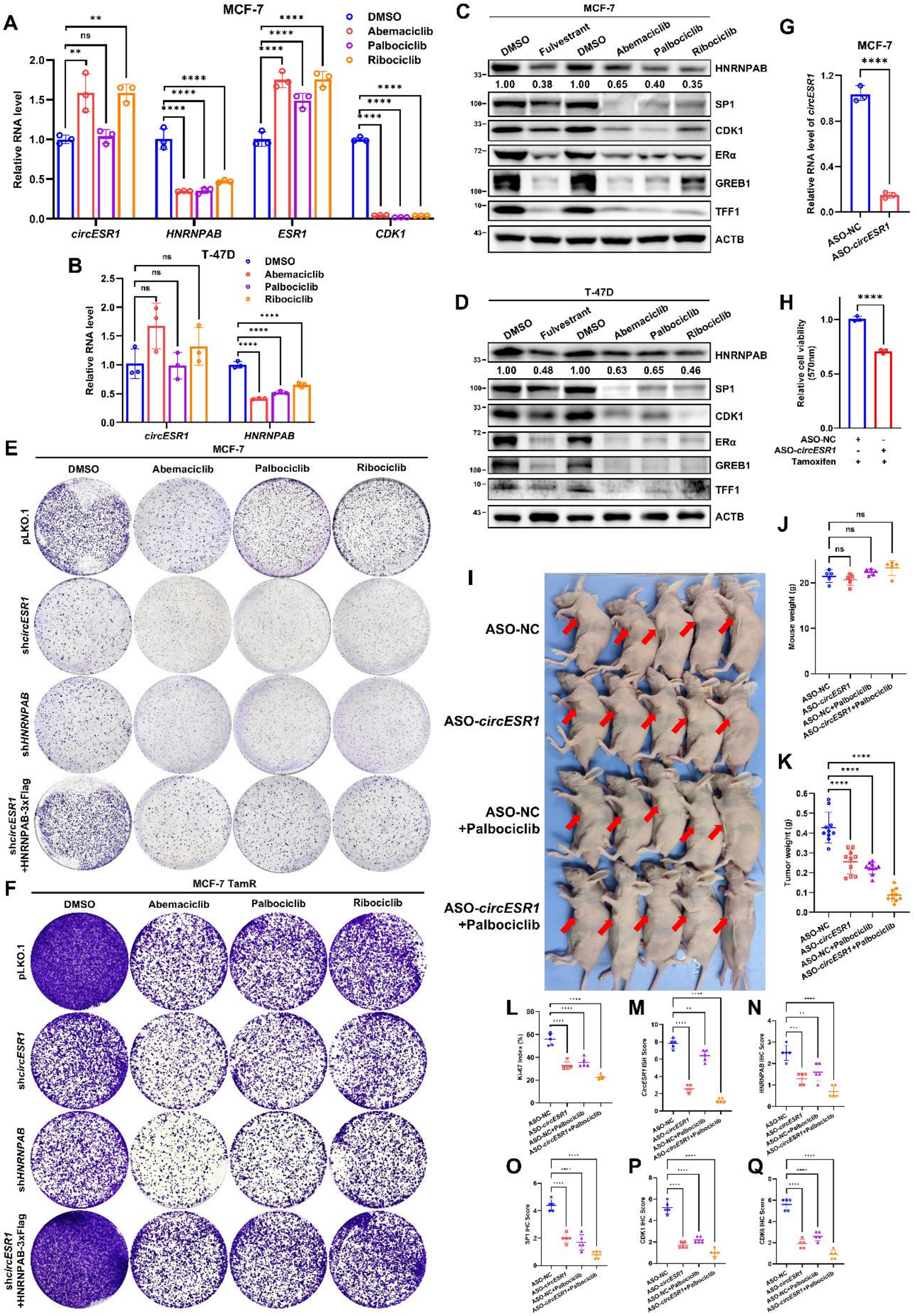

233

Figure S14 Combined treatment of antiestrogen-resistant ER+ BC with ASO targeting c*ircESR1* and CDK4/6i. (A) The relative expression of *circESR1* and *HNRNPAB*, *ESR1, CDK1* mRNAs after treating 1 μM abemaciclib or 2 μM palbociclib or 10 μM ribociclib in MCF-7 cells for 48 h analyzed by qRT-PCR. (B) The relative expression of *circESR1* and *HNRNPAB* mRNA after treating 1 μM abemaciclib or 2 μM palbociclib or 10 μM ribociclib in T-47D cells for 48 h analyzed by qRT-PCR. (C) Immunoblot assessment of HNRNPAB, SP1, CDK1, ERα, GREB1 and TFF1 proteins after treating 1 μM abemaciclib or 2 μM palbociclib or 10 μM ribociclib in MCF-7 cells for 48 h. (D) Immunoblot assessment of HNRNPAB, SP1, CDK1, ERα, GREB1 and TFF1 proteins after treating 1 μM abemaciclib or 2 μM palbociclib or 10 μM ribociclib in T-47D cells for 48 h. (E) Cell viability in MCF-7 cells bearing control, or *circESR1* shRNA, or *HNRNPAB* sgRNA, or *circESR1* shRNA and vector expressing HNRNPAB treating DMSO or 1 μM abemaciclib or 2 μM palbociclib or 10 μM ribociclib determined by colony formation assays. (F) Cell viability in MCF-7 TamR cells bearing control, or *circESR1* shRNA, or *HNRNPAB* shRNA determined by colony formation assays, or *circESR1* shRNA and vector expressing HNRNPAB treating DMSO or 1 μM abemaciclib or 2 μM palbociclib or 10 μM ribociclib. (G) After transient transfection of control ASO or ASO targeting *circESR1* in MCF-7 cells for 48 h, the relative expression of *circESR1* analyzed by qRT-PCR. (H) Cell viability in MCF-7 TamR cells bearing ASO or ASO targeting *circESR1* with treating 1 μM tamoxifen determined by MTT assays. (I-K)

Female BALB/c nude mice injected with 1×10^6 MCF-7 TamR cells (n=5/group). On the 48th day, when collecting tumors from mice, weight of the live mice and isolated tumors were measured. (L) Ki-67 staining in tumor sections of Figure 6K was assessed by H-score, n=5. (M) *CircESR1* ISH score in tumor sections of Figure 6K, n=5. (N-Q) IHC score of HNRNPAB, SP1, CDK1 and CDK6 in tumor sections of Figure 6K, n=5. Data was shown as mean ± S.D. from three independent experiments. Unpaired two-tailed Student's *t* test (G-H) and one-way ANOVA followed by Tukey's multiple comparisons test (A-B, J-K). ns, $P>0.05$; **, $P<0.01$; ****, $P<0.0001$.
